# Monocarboxylate transporter 1 deficiency impacts CD8^+^ T lymphocytes proliferation and recruitment to adipose tissue during obesity

**DOI:** 10.1016/j.isci.2022.104435

**Published:** 2022-05-23

**Authors:** C. Macchi, A. Moregola, M.F. Greco, M. Svecla, F. Bonacina, S. Dhup, R.K. Dadhich, M. Audano, P. Sonveaux, C. Mauro, N. Mitro, M. Ruscica, G.D. Norata

**Affiliations:** 1 Department of Pharmacological and Biomolecular Sciences, Università degli Studi di Milano, Milan, Italy; 2 Pole of Pharmacology, Institut de Recherche Experimentale et Clinique (IREC), Université catholique de Louvain (UCLouvain), Brussels, Belgium; 3 Institute of Inflammation and Ageing, College of Medical and Dental Sciences, University of Birmingham, Birmingham, UK; 4 SISA Center for the Study of Atherosclerosis, Bassini Hospital, Via M. Gorki 50, 20092 Milan, Cinisello Balsamo, Italy

**Keywords:** Biological sciences, Immunology, Metabolomics, Proteomics

## Abstract

Lactate sits at the crossroad of metabolism, immunity, and inflammation. The expression of cellular lactate transporter MCT1 (known as Slc16a1) increases during immune cell activation to cope with the metabolic reprogramming. We investigated the impact of MCT1 deficiency on CD8^+^ T cell function during obesity-related inflammatory conditions. The absence of MCT1 impaired CD8^+^ T cell proliferation with a shift of ATP production to mitochondrial oxidative phosphorylation. In *Slc16a1*^*f/f*^*Tcell*^*cre*^ mice fed a high-fat diet, a reduction in the number of CD8^+^ T cells, which infiltrated epididymal visceral adipose tissue (epiWAT) or subcutaneous adipose tissue, was observed. Adipose tissue weight and adipocyte area were significantly reduced together with downregulation of adipogenic genes only in the epiWAT. Our findings highlight a distinct effect of MCT1 deficiency in CD8^+^ T cells in the crosstalk with adipocytes and reinforce the concept that targeting immunometabolic reprogramming in lymphocyte could impact the immune-adipose tissue axis in obesity.

## Highlights

• MCT1 deficiency derails cell metabolism in activated CD8^+^ T cells
• MCT1 deficiency reduces cell proliferation of activated CD8^+^ T cells
• In obese mice, MCT1 deficiency reduces CD8^+^ T effector cells infiltration in epiWAT
• The lack of MCT1 in CD8^+^ T cells leads to smaller adipocytes with a beiging phenotype

## Graphical abstract

**Figure:**
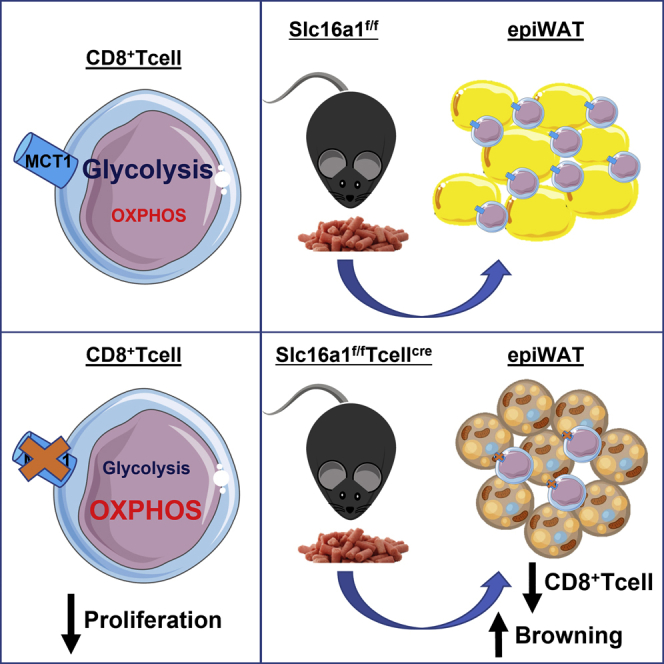

## Introduction

Lactate, considered for a long time a mere waste product of cellular metabolism, has been recognized, in the last decades, as an active molecule capable of modulating the immune response (Pucino et al., 2019). Indeed, far from being inert, lactate accumulation has been described to be at the crossroad of metabolism, immunity, and inflammation (Certo et al., 2021). Lactate production occurs mainly in the cytoplasm under hypoxic conditions or as a consequence of elevated glycolytic flux in proliferating cells. Its intracellular levels are regulated mainly through the carrier function of specific transporters on the cell plasma membrane. Two transporter families have been identified that exert this function, namely, proton-coupled monocarboxylate transporters (MCTs) and sodium-coupled monocarboxylate transporters. Among them, MCT1 (also known as Slc16a1; gene name: *Slc16a1*) has a high specificity for lactate coupled to a broad tissue expression (Broer et al., 1998; Halestrap, 2013).

The function of this transporter is relevant when immune cells and particularly T lymphocyte are activated; indeed, following activation, they undergo a rapid rearrangement in cellular metabolism to support proliferation via an adequate supply of macromolecules (Menk et al., 2018; Norata et al., 2015; Renner et al., 2019). CD4^+^ and CD8^+^ T cell metabolic changes during activation involve a switch from oxidative phosphorylation to aerobic glycolysis (Warburg effect). Despite the availability of oxygen for complete glucose oxidation, glucose is metabolized into lactate, rather than processed via tricarboxylic acid (TCA) cycle and oxidized in mitochondria (Chapman et al., 2020; Gerriets and Rathmell, 2012). Lactate accumulates into the extracellular space, affecting T cell mobility and cytolytic capacity (Haas et al., 2015). The T cell switch from a quiescent state to an activated status upon exposure to inflammatory stimuli is particularly relevant during obesity, where the development of a low-grade inflammatory milieu within white adipose tissue occurs (Hotamisligil, 2017). This condition is characterized by the production of inflammatory mediators by adipocytes and macrophages within the adipose tissue that in turn impacts systemic metabolism (Hotamisligil et al., 1993; Xu et al., 2003). Besides the infiltration of macrophages in white adipose tissue and the shift in macrophages polarization (Lumeng et al., 2007), different immune cells (e.g., T lymphocytes, including CD4^+^, CD8^+^, and Tregs, eosinophils, and neutrophils) have been implicated in obesity-associated metabolic disturbances (Mauro et al., 2017; McLaughlin et al., 2017). Among them, the infiltration of activated T cells such as CD8^+^ effector plays a critical role in the initiation and propagation of obesity-induced inflammation (Nishimura et al., 2009).

On these premises, we aimed at characterizing the role of MCT1 selective deficiency in lymphocyte T cells in modulating T cell effector response during a state of low-grade inflammation. We showed that the selective lack of MCT1 in CD8^+^ T cells affects their metabolic reprogramming as well as their recruitment in adipose tissue during obesity.

## Results

### MCT1-deficient CD8^+^ T cells present impaired proliferation and protein signature following activation

**Figure 1:**
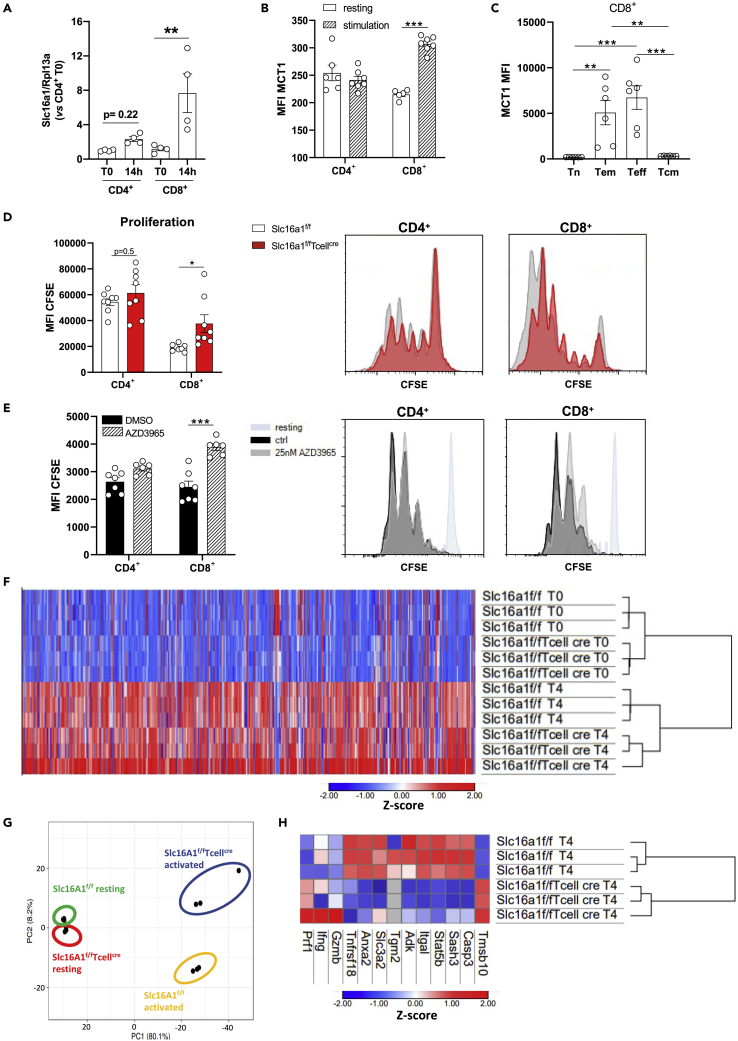
MCT1-deficient CD8^+^ T cells present impaired proliferation and cytokine production (A and B) MCT1 is presented as gene expression (*Slc16a1*) (A) and MFI (B) in CD4^+^ and CD8^+^ T cells isolated from *Slc16a1*^*f/f*^ mice at basal condition and after stimulation. n = 4 per group for gene expression analysis. n = 5–7 per group for FACS analysis. (C) MCT1 protein expression in CD8^+^ T cells subsets after overnight stimulation *in vitro* with anti-CD3 and anti-CD28 assessed by flow cytometry. n = 6 per group. (D) Proliferation of CD4^+^ and CD8^+^ T cells isolated from *Slc16a1*^*f/f*^ and *Slc16a1*^*f/f*^*Tcell*^*cre*^ mice after *in vitro* activation with anti-CD3 and anti-CD28 for 4 days is presented as MFI for CFSE staining. The analysis of CFSE proliferation assays is based on the premise that the label is halved in the two daughter cells. Considering that all the cells receive the same amount of CFSE at the beginning, the cell cycle can be followed by the progressive decrease of dye intensity in the cells. n = 8 per group. (E) Proliferation of CD4^+^ and CD8^+^ T cells isolated from *Slc16a1*^*f/f*^ mice after *in vitro* activation with anti-CD3 and anti-CD28 for 4 days and in presence of DMSO (control) or MCT1 inhibitor, AZD3965, is presented as MFI for CFSE staining. n = 6–7 per group. (F) Heatmap and hierarchical clustering of the proteome of CD8^+^ T cells isolated from *Slc16a1*^*f/f*^ and *Slc16a1*^*f/f*^*Tcell*^*cre*^ mice at basal condition and after *in vitro* activation show relative protein expression values (*Z* score transformed LFQ protein intensities). Data are presented as triplicate for each subset. (G) Principal component analysis of CD8^+^ T cell isolated from *Slc16a1*^*f/f*^ and *Slc16a1*^*f/f*^*Tcell*^*cre*^ mice in basal condition and after *in vitro* stimulation. (H) Heatmap and hierarchical clustering of the proteome of CD8^+^ T cells isolated from *Slc16a1*^*f/f*^ and *Slc16a1*^*f/f*^*Tcell*^*cre*^ mice after *in vitro* activation show relative protein expression value (*Z* score transformed LFQ protein intensities) linked to cell activation and cytokine production. Data are presented as triplicate for each subset. In (A–E) data are presented as mean ± SEM. Differences between groups have been assessed by Mann-Whitney t test or two-way ANOVA. ^∗^p < 0.05, ^∗∗^p < 0.01 versus the respective control. T_em_, T effector memory; T_cm_, T central memory; T_eff_, T effector; T_n_, T naive; CFSE, carboxyfluorescein succinimidyl ester; CTRL, control; FACS, fluorescence-activated cell sorting; LFQ, label free quantified; MCT1, monocarboxylate transporter 1; MFI, median fluorescence intensity; *Slc16a1*, solute carrier family 16 member 1.

In agreement with previous findings (Haas et al., 2015), MCT1 is expressed to a larger extent in CD8^+^ as compared with CD4^+^ T cells. *Slc16a1* mRNA expression increased by 6.4-fold between resting and activated CD8^+^ T cells (p < 0.01), whereas it was of 2.3-fold between resting and activated CD4^+^ T cells (Figure 1A). MCT1 median fluorescence intensity (MFI) was not significantly different between resting and activated CD4^+^ T cells, whereas in activated CD8^+^ T cells, a significant increase compared with resting ones was observed (+42%, p < 0.001; Figure 1B). More in detail, MCT1 expression was higher in T effector memory (T_em_) and T effector (T_eff_) cells (Figure 1C).

To explore whether this increase was relevant for T cell immunometabolic reprogramming and function, we generated a specific transgenic animal model lacking the MCT1 transporter selectively in CD4^+^ and CD8^+^ T cells (*Slc16a1*^*f/f*^*Tcell*^*cre*^). This model was obtained by crossing mice expressing Cre-recombinase selective in lymphocyte T cells with *Slc16a1*^*f/f*^ mice, resulting in the deletion of exons 2 and 3 of the *Slc16a1* gene (Figure S1A) and the deficiency of MCT1 protein in CD4^+^ and CD8^+^ T cells (Figures S1B and S1C).

Because in our murine model the deletion of *Slc16a1* in lymphocytes takes place in the thymus, we ruled out a possible effect on T cells development; there were no alterations in the number of thymic CD8^+^ T cells and double-positive cells (DP, positive for CD4^+^ and CD8^+^), not subjected yet to thymic selection (Figures S2A and S2B). The same was true for their viability (Figures S2C and S2D).

We next tested whether MCT1 deficiency impacts T cells response following activation, namely, CD4^+^ and CD8^+^ T cells from *Slc16a1*^*f/f*^*Tcell*^*cre*^ *and Slc16a1*^*f/f*^ were incubated with anti-CD3, anti-CD28, and interleukin (IL)2 for 4 days. In agreement with the observation that MCT1 expression was significantly increased only in CD8^+^ T cells following activation, MCT1 deficiency largely affected CD8^+^ but not CD4^+^ T cells proliferation. In particular, the relative distribution in the different cell division phases highlights a profile toward a prevalence of more cells that undergo an increased division in wild-type (WT) CD8^+^ T cells as compared with CD8^+^ T cells lacking MCT1 (MCT1 KO CD8^+^ T cells). This profile was not evident in CD4^+^ T cells as assessed by CFSE proliferation assays (Figure 1D) and absolute count (Figure S1D). This finding was confirmed by the use of AZD3965, a MCT1 inhibitor, which did not affect the proliferation of CD4^+^ T cells but decreased that of CD8^+^ T cells (Figure 1E). This evidence confirmed a robust impact of MCT1 deficiency on CD8^+^ T cells but not on CD4^+^ T cells, thus supporting a further characterization of the CD8^+^ T cells compartment in this animal model. Because in T cells, lactate can be also transported by MCT2 and MCT4, we assessed whether the expression of these two transporters could be affected by MCT1 deficiency in activated CD8^+^ T cells. Although for MCT2, no differences were found in mRNA and protein expression in CD8^+^ T cells isolated from *Slc16a1*^*f/f*^ and *Slc16a1*^*f/f*^*Tcell*^*cre*^ mice, a reduced protein, but not mRNA expression, of MCT4 was observed in *Slc16a1*^*f/f*^*Tcell*^*cre*^ mice CD8^+^ T cells compared with their control counterpart (Figures S1E and S1F). Furthermore, MCT1 KO CD8^+^ T cells presented a roughly 3.5-fold less expression of the ancillary CD147 protein compared with WT cells (Figure S1G).

Therefore, we analyzed the proteome of these cells under resting and activated conditions compared with controls (Figure 1F). Intriguingly, although the proteome was similar between resting MCT1 KO CD8^+^ T cells and their control counterpart, a different profile was observed upon activation (Figure 1G). Specifically, MCT1 KO CD8^+^ T cells presented a minor increase in proteins usually upregulated during activation (e.g., Itgal and Tnfrsf18 [Ronchetti et al., 2012]) and an opposite trend in those who are generally reduced after activation (e.g., Tmsb10; Figure 1H). This finding was in line with the reduced proliferation of MCT1 KO CD8^+^ T cells compared with their WT counterpart and with the enrichment pathway analysis, showing that ribosome and spliceosome pathways, both playing a key role in protein synthesis (Figures S1H and S1I), were among the least upregulated following activation in MCT1 KO CD8^+^ T cells. Finally, relative to changes in cytokine production, the overall impact of MCT1 deletion in T cells seemed to affect significantly only IL17, as assessed by MFI (Figure S2E).

### The lack of MCT1 affects energy metabolism of CD8^+^ T cells

**Figure 2:**
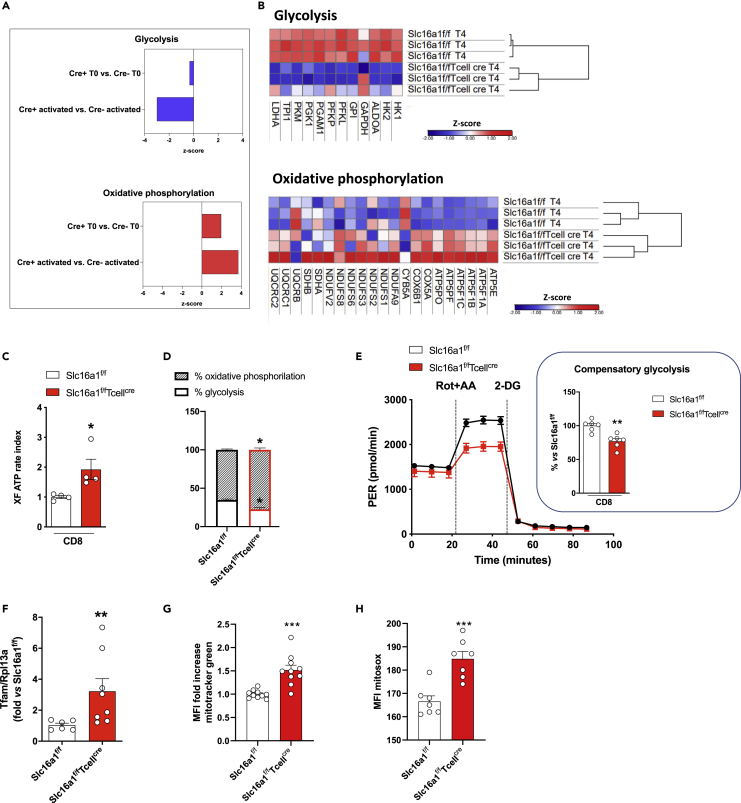
MCT1 deletion impacts CD8^+^ T cell cellular reprogramming (A) Proteomic analysis of metabolic pathways in CD8^+^ T cells isolated from *Slc16a1*^*f/f*^ and *Slc16a1*^*f/f*^*Tcell*^*cre*^ mice after *in vitro* activation with anti-CD3 and anti-CD28 for 4 days. Blue bars indicate glycolysis, and red bars indicate oxidative phosphorylation. (B) Heatmap and hierarchical clustering of the proteome of CD8^+^ T cells isolated from *Slc16a1*^*f/f*^ and *Slc16a1*^*f/f*^*Tcell*^*cre*^ mice after *in vitro* activation show relative protein expression values (*Z* score transformed LFQ protein intensities) for glycolysis and oxidative phosphorylation; data are presented as triplicate for each subset. (C–E) Measurement of (C, D) ATP production from mitochondrial respiration and glycolysis and of (E) glycolysis in CD8^+^ T cells isolated from *Slc16a1*^*f/f*^ and *Slc16a1*^*f/f*^*Tcell*^*cre*^ mice after *in vitro* activation with anti-CD3 and anti-CD28 for 4 days; n = 5 per group. (F) Gene expression of transcription factor A mitochondrial (Tfam) in CD8+ T cells isolated from *Slc16a1f/f* and *Slc16a1f/fTcellcre* mice after *in vitro* activation with anti-CD3 and anti-CD28 for 4 days. n = 3–4 per group. (G) Mitotracker green median fluorescence intensity (MFI, fold increase) in CD8^+^ T cells isolated from *Slc16a1*^*f/f*^ and *Slc16a1*^*f/f*^*Tcell*^*cre*^ mice after *in vitro* activation with anti-CD3 and anti-CD28 for 4 days; n = 10 per group. (H) Mitosox fluorescence (MFI) in CD8^+^ T cells isolated from *Slc16a1*^*f/f*^ and *Slc16a1*^*f/f*^*Tcell*^*cre*^ mice after *in vitro* activation with anti-CD3 and anti-CD28 for 4 days; n = 7 per group. In (C–H) data are presented as mean ± SEM. Differences between groups have been assessed by unpaired two-side t test. ^∗^p < 0.05, ^∗∗^p < 0.01, ∗∗∗p < 0.001 versus respective control. AA, antimycin A; LFQ, label free quantified; MFI, median fluorescence intensity; PER, proton efflux rates; Rot, Rotenone; 2-DG, 2-deoxy-D-glucose; Slc16a1, solute carrier family 16 member 1.

T cells exit from quiescence state is associated with anabolic metabolism and the reprogramming of mitochondrial metabolism (Chapman et al., 2020). This adaptation appears to be less effective in activated MCT1 KO CD8^+^ T cells, which showed a shift between glycolysis and oxidative phosphorylation, as compared with activated MCT1 CD8^+^ T cells (Figure 2A).

When comparing KO cells, an overall decrement of principal glycolytic enzymes, including aldolase A (ALDOA), phosphoglycerate kinase 1 (PGK1), hexokinase (HK), lactate dehydrogenase (LDH), and pyruvate kinase (PKM), was found (Figure 2B). Conversely, mitochondrial electron transport chain complexes components, and the expression of NADH:ubiquinone oxidoreductase core subunits (e.g., NDUFS1, NDUFS2, NDUFS3, NDUFS8, NDUFS9) or ubiquinol-cytochrome C reductase core proteins (e.g., UQCRC1, UQCRC2) together with ATP synthases (e.g., ATP5E, ATP5F1A, ATP5PF, ATP5PO), were increased (Figure 2B).

Moreover, MCT1 KO CD8^+^ T cells relied more on mitochondrial oxidative phosphorylation than on glycolysis to produce ATP (+12% of the oxidative phosphorylation and −12% of glycolysis; both p < 0.05; Figures 2C and 2D). This finding was accompanied by a reduced compensatory glycolysis following the administration of rotenone and antimycin A (−23%; p < 0.01) (Figure 2E). This shift toward oxidative phosphorylation preference under MCT1 deficient conditions could be the consequence of increased mitochondrial function. In line with this hypothesis, we investigated changes in mitochondrial biogenesis and function in CD8^+^ T cells isolated from *Slc16a1*^*f/f*^*Tcell*^*cre*^ and *Slc16a1*^*f/f*^ mice and activated with antiCD3 and antiCD28 antibodies for 96 h. The expression of the key transcription factor involved in mitochondrial biogenesis (mitochondrial transcription factor A—Tfam) was significantly increased in activated *Slc16a1*^*f/f*^*Tcell*^*cre*^ mice CD8^+^ T cells compared with those of *Slc16a1*^*f/f*^ mice (Figure 2F). Accordingly, mitochondrial mass and mitochondrial oxidative function were increased in activated *Slc16a1*^*f/f*^*Tcell*^*cre*^ mice CD8^+^ T cells compared with those of *Slc16a1*^*f/f*^ mice (Figures 2G and 2H).

**Figure 3:**
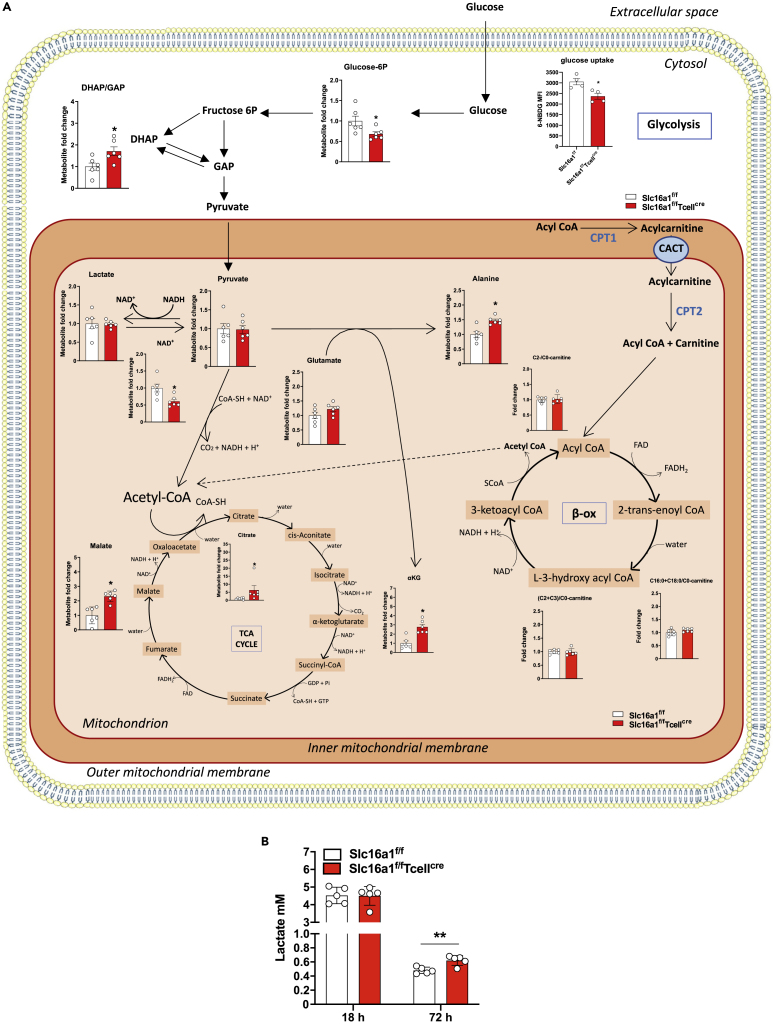
MCT1 deletion impacts CD8^+^ T cell metabolome reprogramming (A) Scheme of metabolome reprogramming and (B) extracellular levels of lactate (mM) in CD8^+^ T cells isolated from *Slc16a1*^*f/f*^ and *Slc16a1*^*f/f*^*Tcell*^*cre*^ mice after *in vitro* activation with anti-CD3 and anti-CD28 for 4 days n = 6 per group. Data are presented as mean ± SEM, and differences between groups were assessed by Fisher’s Least Significant Difference (LSD) test with false discovery rate (FDR) correction. ^∗^FDR <0.1 versus respective control (A). Differences have been evaluated by Mann-Whitney t test (B).

To strengthen these findings, we profiled the amount of energy metabolites, including those related to glycolysis, Krebs cycle acylcarnitines, cofactors, and free amino acids by targeted metabolomics. Although the metabolomic profile was similar between MCT1 KO CD8^+^ T cells and their WT counterpart under basal conditions (Figure S3A), upon activation, MCT1 deficiency led to significant differences in the metabolomic profile (Figure S3B). Roughly 20% of metabolites detected were downregulated and 17% upregulated in MCT1 KO CD8^+^ T cells (Figure S3C). As shown in Figure 3A, among glycolysis intermediates, we observed reduced levels of glucose-6P in line with the decreased uptake of 6-NBDG, an analogue of glucose, which indirectly marks increased or decreased glycolytic activity (de Kivit et al., 2020). An increase in dihydroxyacetone-P/glyceraldehyde-3P (DHAP/GAP) ratio and an accumulation of alanine were also observed. These findings, together with the absence of major changes in pyruvate and lactate, point to a reduced glycolytic flux paralleled by the upregulation of pyruvate transamination to alanine in MCT1 KO CD8^+^ T cells compared with WT cells (Figure 3A). In line with this evidence, a significant reduction of NAD^+^ levels in MCT1 KO CD8^+^ T cells was found.

Furthermore, we found increased levels of proline (Figure S3D) and α-ketoglutarate (αKG) together with malate and citrate in MCT1 KO CD8^+^ T cells, which provide further evidence that when MCT1 is deleted in CD8^+^ T cells, pyruvate is transaminated to alanine as well as redirected to TCA cycle (Figure 3A). In line with this, urea cycle intermediates, ornithine and citrulline, were significantly downregulated in MCT1 KO CD8^+^ T cells compared with WT, together with several acylcarnitines (which mark fatty acid β-oxidation activity), such as acetylcarnitine (C2), propionylcarnitine (C3), butyrylcarnitine (C4), and octadecanoylcarnitine (C18:0) (Figures S3D and 3A). Moreover, we did not observe differences in acetyl-carnitine (C2)/free-carnitine (C0) ratio or acetyl-carnitine (C2)+propionyl-carnitine/free-carnitine (C0) ratio (C2+C3)/C0; indexes of even and overall β-oxidation efficiency, respectively (Figure S3E). In addition, we did not detect any difference in (C16+C18)/C0-carnitine ratio, suggesting no differences in the activity of the enzyme carnitine palmitoyl-transferase 1A (Figure S3E). Together, these data suggest an overall decrease of the β-oxidation flux, rather than a reduction in the efficiency. Indeed, we observed decreased levels of free carnitine, C2-acylcarnitine, C3-acylcarnitine, C4-acylcarnitine, and C18:0-acylcarnitine (Figure 3A), which indicates that an overall impairment of fatty acid β-oxidation could be present in MCT1 KO CD8^+^ T cells. To corroborate this hypothesis, we analyzed α-ketoglutarate/glutamate ratio; this was significantly upregulated, suggesting that the conversion of glutamate to α-ketoglutarate is favored in MCT1 KO CD8^+^ T cells (Figure S3F). We also analyzed α-ketoglutarate/citrate ratio to evaluate if glutamate, rather than other TCA cycle precursors, may lead to increased α-ketoglutarate levels in MCT1 KO CD8^+^ T cells. Notably, α-ketoglutarate/citrate was unchanged when compared with control, suggesting that mainly glutamate to α-ketoglutarate conversion is upregulated in MCT1 KO CD8^+^ T cells (Figure 3F).

We measured also extracellular lactate levels in activated CD8^+^ T cells from *Slc16a1*^*f/f*^ and *Slc16a1*^*f/f*^*Tcell*^*cre*^ mice; although no differences were observed between WT and MCT1 KO CD8^+^ T cells after 18 h (4.513 and 4.50 mM, respectively), a significant increase in lactate levels was observed after 72 h in the supernatant of MCT1 KO CD8^+^ T cells *versus* WT (0.6214 and 0.481 mM, respectively; Figure 3B). These results may lead to hypothesize that (1) MCT1 is mainly involved in lactate import in these cells and (2) that lactate may represent an important energy fuel upon T cell activation (Figures 3A and 3B). This is in line with other reports where lactate was demonstrated to be used as an energy source in several lymphoid lineages (Caslin et al., 2021; Watson et al., 2021). These data, coupled to the metabolomic profiling of other pathways, could suggest that CD8^+^ T cells lacking MCT1 still handle lactate differently and sustain energy production by alternative sources such as the glutamine pathway.

### Immuno-metabolic signature of obese mice selectively lacking MCT1 in T cells

Beyond innate immunity, it is emerging how the adaptive immune system plays a key role during obesity (McLaughlin et al., 2017); we therefore focused our attention on the potential role of MCT1 in CD8^+^ T cell response during obesity.

**Figure 4:**
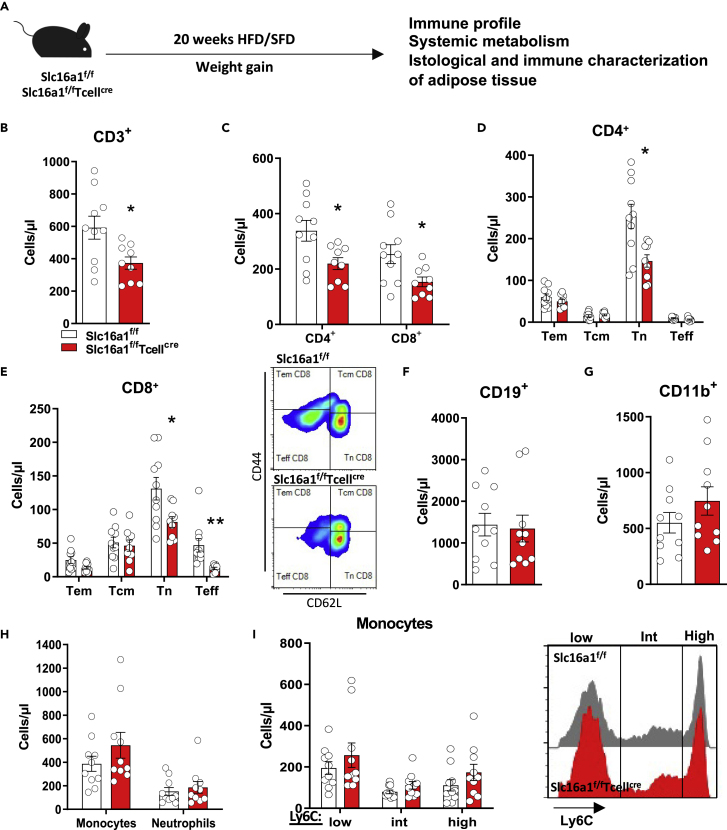
Immunophenotypic signature upon high-fat diet (A) Scheme of the experimental plan. (B–I) Systemic immune cell profile of *Slc16a1*^*f/f*^ and *Slc16a1*^*f/f*^*Tcell*^*cre*^ mice fed an HFD for 20 weeks. Adaptive immune compartement is represented by T lymphocytes (B, CD3^+^ cell), CD4^+,^ and CD8^+^ lymphocytes (C), CD4^+^ (D) and CD8^+^ subsets (E) (T effector memory—Tem, T central memory—Tcm, T naive—Tn, T effector—Teff), and by B lymphocytes (F, CD19^+^ cell). n = 9–10 per group. Innate immune cells are represented by CD11b positive cells (G), monocytes, neutrophils (H), and monocytes subsets based on Ly6C positivity (I). n = 10 per group. In (B–I) data are presented as mean ± SEM. Differences between groups have been assessed by unpaired two-side t test or by two-way ANOVA or Kruskal-Wallis nonparametric test. ^∗^p < 0.05, ^∗∗^p < 0.01 versus respective controls. HFD, high-fat diet; SFD, standard-fat diet; Slc16a1, solute carrier family 16 member 1; Int, intermediate.

To this aim, *Slc16a1*^*f/f*^*Tcell*^*cre*^ mice and their control counterpart (*Slc16a1*^*f/f*^) were fed either a high-fat diet (HFD) or a standard-fat diet (SFD) for 20 weeks (Figure 4A). Regardless of the genotype, exposure to HFD resulted in the significant increase in weight gain, glycemia, as well as in impaired glucose tolerance and insulin sensitivity (Figures S4A–S4C). Cholesterol and triglycerides levels were comparable between the two groups (Figures S4D and S4E). Interestingly, a lower number of circulating T lymphocytes (CD3^+^ cells) was found in *Slc16a1*^*f/f*^*Tcell*^*cre*^ mice compared with *Slc16a1*^*f/f*^ mice fed HFD (Figure 4B), with a reduction in the number of CD4^+^ and CD8^+^ T cells (Figure 4C), naive T (T_n_) CD4^+^ and CD8^+^ T cells, and T_eff_ CD8^+^ T cells (Figures 4D and 4E). B lymphocytes (CD19^+^), CD11b cells, monocytes, neutrophils, and monocyte-associated subsets (ly6C low, intermediate, and high) were similar between groups (Figures 4F–4I). The analysis of transcription factors of T cells resilient into epididymal white adipose tissue (epiWAT) of mice fed an HFD did not show significant differences between *Slc16a1*^*f/f*^ and *Slc16a1*^*f/f*^*Tcell*^*cre*^ mice for Gata3 and T-bet in both CD8^+^ and CD4^+^ T cells or CD8^+^ T perforin content (Figure S5A). Conversely, although a reduction in both plasma and epiWAT tumor necrosis factor (TNF)-α levels was observed in *Slc16a1*^*f/f*^*Tcell*^*cre*^ mice compared with *Slc16a1*^*f/f*^ mice, this difference reached a statistical significance only for plasma levels (Figure S5B).

### Selective lack of MCT1 in CD8^+^ cells affects epiWAT milieu

Because CD8^+^ T cells increase in the adipose tissue of both diet-induced or genetic obese mice (Rausch et al., 2008), we explored whether the selective lack of MCT1 in CD8^+^ T cells influenced their accumulation in adipose tissue, including epiWAT and subcutaneous (SCAT) adipose tissues. As expected, HFD led to a significant increase of CD8^+^ T cells infiltrating the epiWAT, in particular, the T_em_ subpopulation (Figure S5C). These findings were not as robust in SCAT where the number of infiltrating T cells and their effector subsets were not statistically different between SFD- and HFD-fed mice (Figure S5D).

**Figure 5:**
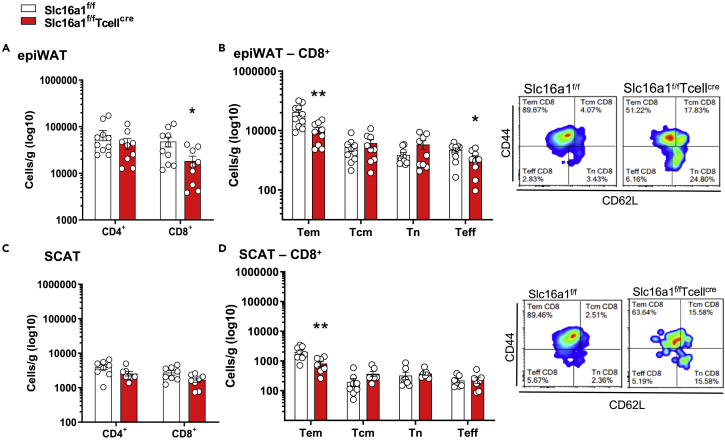
Immunophenotypic characterization of epididymal and subcutaneous adipose tissues in mice fed HFD (A and B) Flow cytometric analysis of epiWAT T lymphocytes and CD8^+^ subpopulations in *Slc16a1*^*f/f*^ and *Slc16a1*^*f/f*^*Tcell*^*cre*^ mice fed an HFD for 20 weeks; n = 7 per group. (C and D) Flow cytometric analysis of SCAT T lymphocytes and CD8^+^ T subpopulations in *Slc16a1*^*f/f*^ and *Slc16a1*^*f/f*^*Tcell*^*cre*^ mice fed an HFD for 20 weeks; n = 7 per group.In (A–D) data are presented as mean ± SEM. Differences between groups have been assessed by unpaired two-side t test or by two-way ANOVA or Kruskal-Wallis nonparametric test. ^∗^p < 0.05, ^∗∗^p < 0.01 versus respective control; epiWAT, epididymal white adipose tissue; SCAT, subcutaneous adipose tissue; HFD, high-fat diet; Slc16a1, solute carrier family 16 member 1.

The immunophenotipic characterization of epiWAT and SCAT, in mice fed HFD for 20 weeks, showed that MCT1 deficiency in T cells resulted in a significant reduction of the number of CD8^+^ T cells, which infiltrated either epiWAT or SCAT (Figures 5A–5D). In more details, T_em_ were reduced both in epiWAT and in SCAT; instead, T_eff_ were reduced only in epiWAT (Figures 5E and 5F). These differences were not the consequence of increased CD8^+^ T cells death in the adipose tissue of *Slc16a1*^*f/f*^*Tcell*^*cre*^ animals, as the percentage of dying CD8^+^ T cells was similar between the two genotypes (Figures S5E and S5F). No significant differences in the number of CD4^+^ T cells were found in both epiWAT and SCAT (Figures 5A and 5C). Moreover, in mesenteric lymphnodes the number of CD4^+^ T and CD8^+^ T cells were similar among animal models (Figures S6A–S6C). These findings suggest that MCT1 deficiency impacted primarily epiWAT T cells recruitment under HFD regimen. The observation of a similar number of CD11b^+^ cells in epiWAT and SCAT also excluded a major contribution of macrophages and monocytes on the phenotype observed in *Slc16a1*^*f/f*^*Tcell*^*cre*^ mice (Figures S6D–S6G).

**Figure 6:**
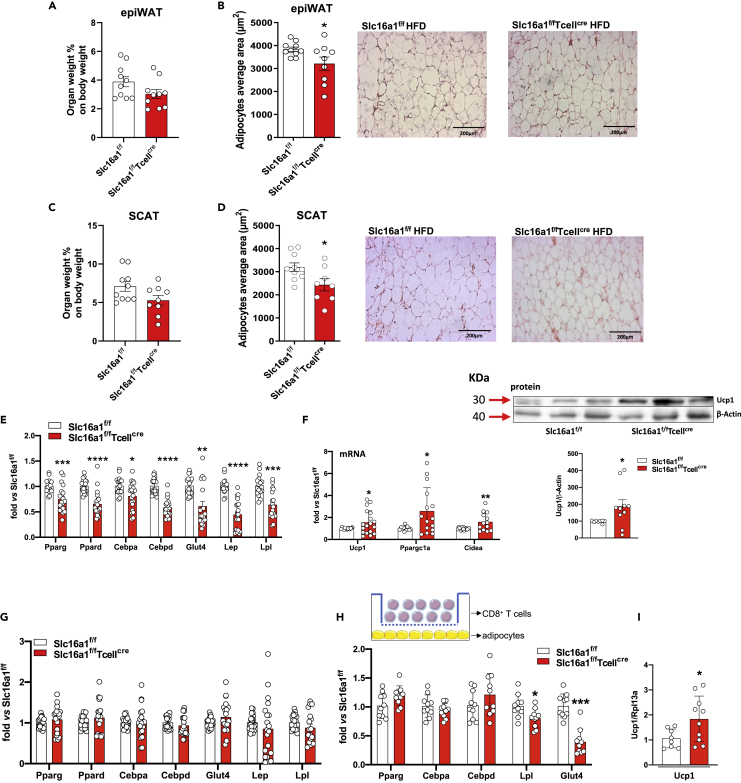
T cell deletion of Slc16A1 affects epiWAT adipocyte area and adipogenesis (A and B) Percentage of weight of epiWAT (A) and quantification of adipocytes area (B) in *Slc16a1*^*f/f*^ and *Slc16a1*^*f/f*^*Tcell*^*cre*^ mice fed an HFD for 20 weeks; n = 6–10 per group. (C and D) Percentage of weight of SCAT (C) and quantification of adipocytes area (D) in *Slc16a1*^*f/f*^ and *Slc16a1*^*f/f*^*Tcell*^*cre*^ mice fed an HFD for 20 weeks; n = 6–10 per group. (E) Expression of adipogenic genes in epiWAT of *Slc16a1*^*f/f*^ and *Slc16a1*^*f/f*^*Tcell*^*cre*^ mice fed an HFD for 20 weeks; n = 9–10 per group. (F) Gene and protein expression of UCP1 and gene expression of PGC1α and Cidea in epiWAT of *Slc16a1*^*f/f*^ and *Slc16a1*^*f/f*^*Tcell*^*cre*^ mice fed an HFD for 20 weeks; n = 9–10 per group. (G) Expression of adipogenic genes in SCAT of *Slc16a1*^*f/f*^ and *Slc16a1*^*f/f*^*Tcell*^*cre*^ mice fed an HFD for 20 weeks; n = 9–10 per group. (H) Gene expression of adipogenic genes in co-cultures between MCT1 KO CD8^+^ T cells and murine adipocytes (NIH3T3cells). n = 5 per group. (I) Gene expression of UCP1 in co-cultures between MCT1 KO CD8^+^ T cells and murine adipocytes (NIH3T3cells). n = 4–5 per group.In (A–H) data are presented as mean ± SEM. Differences between groups have been assessed by unpaired two-side t test, Mann-Whitney, or by two-way ANOVA. ^∗^p < 0.05, ^∗∗^p < 0.01, ^∗∗∗^p < 0.001, ^∗∗∗∗^p < 0.0001 versus respective control. Cebp, CCAAT/enhancer binding protein; Cidea, cell-death-inducing DFFA-like effector a; Glut4, glucose transporter type 4; Lep, leptin; Lpl, lipoprotein lipase; Ppar, peroxisome proliferator-activated receptor; Ppargc1a, Pparg coactivator 1 alpha; Rpl13a, ribosomal protein L 13a; Ucp1, uncoupling protein 1; epiWAT, epididymal white adipose tissue; SCAT, subcutaneous adipose tissue; HFD, high-fat diet; Slc16a1, solute carrier family 16 member 1 (monocarboxylic transporter 1).

To evaluate if the reduced infiltration of CD8^+^ cells in *Slc16a1*^*f/f*^*Tcell*^*cre*^ mice could impact adipose tissue biology, we extensively analyzed adipose tissue characteristics. In epiWAT, adipose tissue weight showed a trend toward decrease, whereas the adipocyte area was significantly decreased in *Slc16a1*^*f/f*^*Tcell*^*cre*^ mice compared with their control counterpart (Figures 6A and 6B). A similar trend was observed in SCAT (Figures 6C and 6D).

These results pointed toward a potential crosstalk between CD8^+^ T cells and adipocytes and led us to assess whether the reduction of infiltrating CD8^+^ T cells could affect adipocyte differentiation. The expression of key genes of adipogenesis, namely, CCAAT-enhancer-binding proteins δ (C/EBPδ), peroxisome proliferator-activated receptor γ (PPARγ), C/EBPα, PPARδ, glucose transporter 4 (GLUT4), leptin, and lipoprotein lipase (LPL), was significantly downregulated in epiWAT of *Slc16a1*^*f/f*^*Tcell*^*cre*^ mice fed HFD compared with *Slc16a1*^*f/f*^ mice (Figure 6E). This profile was paralleled by an increase in gene and protein expression of uncoupling protein 1 (UCP1), a marker of browning. In line with these findings, the gene expression of PPARγ coactivator 1 α (PGC-1α) and cell-death-inducing DFFA-like effector a (CIDEA) were also increased (Figure 6F). These differences were peculiar to epiWAT, as no differences in expression of adipogenesis markers were observed in SCAT (Figure 6G).

*In vitro* data on co-cultures between MCT1 KO CD8^+^ T cells and murine adipocytes (NIH3T3cells) supported these findings (Figure 6H). As shown in Figure 6H, NIH3T3cells exposed to CD8^+^ T cells lacking MCT1 presented a reduced expression of GLUT4 and LPL mRNA levels compared with their counterpart, whereas the expression of UCP1 was raised (Figure 6I). No changes were found in markers of early adipocyte differentiation such as PPARγ, c/EBPδ, and c/EBPα, in line with the experimental set-up that exposed NIH3T3cells to activated CD8^+^ T cells at day 6 of differentiation for 72 h (day 9; Figure 6H).

## Discussion

The present study demonstrates that MCT1 transporter deficiency affects metabolic reprogramming of activated CD8^+^ T cells and their recruitment in adipose tissue during obesity. These findings are of note, considering that T cells metabolism, which is highly dynamic, has a tremendous impact on the ability of these cells to proliferate, activate, and differentiate (Gerriets and Rathmell, 2012).

Upon activation, T cells undergo a metabolic switch from oxidative phosphorylation to aerobic glycolysis (Warburg effect) to maintain adequate supply of macromolecules during growth (Gerriets and Rathmell, 2012). Activated T cells prefer to use aerobic glycolysis to rapidly generate ATP, which is accompanied by the concomitant production of lactate (Haas et al., 2013). Although lactate and other abundant monocarboxylic acids, such as pyruvate, play a key role in carbohydrate, fat, and amino acid metabolism (Halestrap, 2012), cells have acquired the capability to eliminate the excess of lactate through specific transporters belonging to the MCT family. Because of its major role in metabolism, L-lactate is quantitatively by far the most important substrate for MCT1, a monocarboxylate transporter that operates equally well in either direction, namely, influx and efflux (Halestrap, 2013). The expression of MCT1 is increased during T-lymphocyte activation and proliferation (Murray et al., 2005), as a consequence of the switch of T-lymphocytes, once activated, to aerobic glycolysis, which results in a massive production of lactate (Vaupel and Multhoff, 2021) and increased lactic acid efflux. Considering that in CD4^+^ T cells the impact of lactate is mediated by Slc5a12 transporter and not by the MCT1 (Haas et al., 2015), we therefore asked whether MCT1 deficiency could impact immunometabolic reprogramming in CD8^+^ T cells. Our data show how interfering with T cell lactate flow across the cell membrane impacts their metabolic adaptation during activation, leading to a reduced glycolytic flux. In support of this finding, we showed that CD8^+^ T cells lacking MCT1 present a reduction in the expression of enzymes governing the glycolytic pathway (i.e., GPI, ALDOA, TPI1, GAPDH, PGK1, PGAM1, and PKM); of note, we observed lower levels of glucose-6-P, whereas DHAP to GAP ratio was increased.

The finding that pyruvate and lactate levels were unaffected while alanine increased suggests that MCT1 CD8^+^ KO T cells favor pyruvate transamination to alanine. To corroborate this hypothesis, we analyzed glutamate and αKG levels, which are a substrate and a product, respectively, of pyruvate transamination. Our data show that αKG levels were increased in MCT1 CD8^+^ KO T cells, whereas glutamate levels were unchanged. Consistently, aspartate was unaffected by MCT1 deletion (Figure 3D), indicating that glutamate utilization is mainly diverted to the transamination of pyruvate to alanine at the expense of the transamination of oxaloacetate to aspartate. Overall, our data would support the hypothesis that in our experimental conditions, T cells could re-arrange their metabolic circuits favoring glutamate conversion to α-ketoglutarate to fuel TCA cycle. Furthermore, the reduced levels of NAD^+^ that we found could be due to increased TCA and OXPHOS activity in MCT1 CD8^+^ KO T cells (Quinn et al., 2020). In line with these findings, we observed higher levels of citrate and malate as well as an increased expression of the mitochondrial OXPHOS complexes, as shown by the upregulation of NADH:CoQ oxidoreductase enzyme (complex I), succinate dehydrogenase (complex II), ubiqynol-cytochrome-c reductase (complex III), cytochrome *c* oxidase (complex IV), and ATP synthase (complex V) subunit expression (Fernandez-Vizarra and Zeviani, 2021). These changes were then reflected in the bioenergetic profile of CD8^+^ T cells lacking MCT1, which showed an increase in the ratio between the fraction of ATP produced by mitochondrial OXPHOS and the fraction derived from glycolysis. Interestingly, MCT1 KO CD8^+^ T cells were not able to properly use glycolysis to compensate for the impaired mitochondrial respiration, further pointing to a feedback mechanism limiting the efficiency of glycolysis. Notably, we also observed reduced levels of ornithine and citrulline, as well as of several acylcarnitines, namely C0 (free), C2-, C3-, C4-, and C18:0 carnitines, thus suggesting a downregulation of urea cycle and fatty acid β-oxidation. Consistently, urea cycle modulation could be dysregulated in CD8^+^ T cells with altered mitochondrial function (Champagne et al., 2016), and fatty acid oxidation in mitochondria could play a role in CD8^+^ T cells activation (Chowdhury et al., 2018; Wang and Green, 2012). According to the evidence that pharmacological inhibition of MCT1 leads to a small but significant reduction in glycolytic flux (Murray et al., 2005), we showed that MCT1 deletion impacted CD8^+^ T cell reprogramming following activation with a shift toward the preference of oxidative phosphorylation to generate ATP. However, this metabolic switch is not sufficient to sustain the anabolic requirements of CD8^+^ T cells, and indeed CD8^+^ T cells lacking MCT1 present a reduced cell proliferation rate compared with controls. Beyond contributing to explain the immunosuppressant potential of the MCT1 antagonist AR-C155858 (Murray et al., 2005), our findings support the hypothesis that MCT1 deficiency could affect CD8^+^ T cells reprogramming during metabolic disturbances such as obesity. Previous studies demonstrated that diclofenac, an MCT1-and MCT4-inhibitor, used as an antitumor agent, does not affect T cell antitumor functions (Renner et al., 2019); in accordance, our paper pointed out a switch in the metabolic pattern and the proliferation of MCT1 KO CD8^+^T cells compared with WT CD8^+^ T cells, without major changes in cytokines production, except for interleukin-17 (IL-17). Furthermore, although different studies showed that MCT1/2 inhibition can be counterbalanced by increased MCT4 expression, this mechanism does not appear to be relevant in our experimental setting (Baek et al., 2014; Marchiq et al., 2015).

The relationship between alterations in adaptive immune cell function in adipose tissue and their contribution to worsen the metabolic phenotype remains an open question. Specifically, the accumulation of pro-inflammatory CD8^+^ T cells in metabolic tissues at very early stages of obesity seems to be crucial for the initiation of obesity-induced inflammation (Wang et al., 2021). CD8^+^ T-cell-deficient mice fed a high-fat diet exhibited a decrease in the inflammatory response, which was restored by adoptive transfer of CD8^+^ T cells. CD8^+^ T cells infiltrating the epiWAT were shown to contribute to the recruitment, differentiation, and activation of macrophages (Nishimura et al., 2009). In line with this evidence, the inhibition of T cell activation limited CD8^+^ T cell and pro-inflammatory macrophages accumulation in adipose tissue of obese mice (Montes et al., 2013). However, it might be speculated that although CD8^+^ T cell infiltration was reduced in both epiWAT and in SCAT (with a difference being significant only in the epiWAT), given that the number of CD8^+^ T cells infiltrating epiWAT for each gram of tissue is approximately 10 times higher compared with those infiltrating SCAT, changes in lymphocyte immune infiltration in epiWAT would have a larger effect on local immune inflammation as could be the case for T cell MCT1-deficient mice. In line with this finding, both CD8^+^ T_eff_ cells and CD8^+^ T_em_ cells were significantly reduced in epiWAT of *Slc16a1*^*f/f*^*Tcell*^*cre*^ mice compared with *Slc16a1*^*f/f*^, whereas only CD8^+^ T_em_ were affected in SCAT.

Of note, MCT1^+/−^ mice exhibit resistance to diet-induced obesity as well as to glucose intolerance and insulin resistance (Lengacher et al., 2013), thus suggesting to further evaluate the role of selective MCT1 deficiency in CD8^+^ T cells during obesity. When mice were fed an obesogenic diet, in spite of a similar weight gain and insulin response in *Slc16a1*^*f/f*^*Tcell*^*cre*^ compared with *Slc16a1*^*f/f*^ mice, thus excluding a major role for MCT1 T cell deficiency on systemic metabolism, a reduced number of CD8^+^ T cells was detected in the epiWAT of *Slc16a1*^*f/f*^*Tcell*^*cre*^ compared with *Slc16a1*^*f/f*^ mice coupled to smaller adipocytes. A possible mechanism beyond this feature could owe to the reduced expression of IL-17 found in CD8^+^ T cells isolated from *Slc16a1*^*f/f*^*Tcell*^*cre*^ mice. Indeed, the suppression of IL-17 axis, at least in mice, promotes adipose-tissue browning, thermogenesis, and energy expenditure (Teijeiro et al., 2021). In line with this observation, although the role of tumor necrosis factor alpha (TNF-α) in obesity-related visceral adipose tissue inflammation remains controversial (Anto Michel et al., 2018; Osborn and Olefsky, 2012), the decrement we found in circulating levels of TNF-α could further contribute to the adipose tissue remodeling between the two animal models. Overall, smaller adipocytes are often correlated with improved metabolic phenotype (Lonn et al., 2010), and in the context of obesity, smaller adipocytes can be associated with a “healthy” phenotype (Vishvanath and Gupta, 2019).

During obesity, adipose tissue expands via adipocyte hypertrophy or via the formation of new adipocytes through differentiation of resident precursors (adipocyte hyperplasia) (Ghaben and Scherer, 2019; Sun et al., 2011). HFD-induced adipose tissue expansion occurs mainly by hypertrophy during the first month of diet, whereas a more prolonged period of HFD feeding (namely, two months or more) increased adipogenesis, which appears to be highly relevant in gonadal adipose tissue (Wang et al., 2013). Vice versa, subcutaneous adipose tissues maintained an extremely low rate of adipogenesis, even after long-term HFD feeding (Wang et al., 2013). The observation that *Slc16a1*^*f/f*^*Tcell*^*cre*^ mice changes in epiWAT adipocyte size is coupled with a downregulation of adipogenic genes and an increase in UCP1 expression further strengthens the hypothesis of a beneficial impact of reducing CD8^+^ T cell recruitment on adipose tissue biology.

In conclusion, our findings highlight a distinct effect of MCT1 deficiency in CD8^+^ T cells on adipocytes, supporting the concept that reprogramming of lymphocytes could impact the immune-adipose tissue axis in the context of obesity without a major impact of systemic metabolism. Indeed, as showed by Lin et al. (2022), the specific deletion of *Slc16a1* in adipose tissue can elicit a vicious cycle of inflammatory response via the functional interaction between adipocytes and macrophages and likely other immune cells, e.g., in our case in which CD8^+^ T cells were involved.

### Limitations of the study

We have to acknowledge some limitations of our study. First, data on lactate kinetics were studied at two specific time points. An evaluation also in a shorter time frame and the use of ^13^C-lactate would have helped in understanding the flux of lactate carbons into acetylCoA or citrate, thus allowing to quantify the amount of lactate used as an energy substrate to fuel TCA cycle. Second, although our data suggest that MCT2 and MCT4 might partially compensate for MCT-1 deficiency, this needs to be evaluated with selective experiments in CD8^+^ T cells with global MCTs deficiency, a condition that might perhaps not be compatible with living cell physiology.

## STAR★Methods

### Key resources table

**Table:** 

REAGENT OR RESOURCE	SOURCE	IDENTIFIER
**Antibodies**		

Mouse monoclonal anti-CD45 PE	BD Bioscience	Cat: 553081; RRID: AB_394611
Mouse monoclonal anti-CD11b AF700	eBioscience	Cat: 56-0112-80; RRID: AB_657586
Mouse monoclonal anti-Ly6C ef450	Invitrogen	Cat: 48-5932-82; RRID: AB_10805519
Mouse monoclonal anti-CD19 PE-Cy7	Invitrogen	Cat: 25-0193-82; RRID: AB_657663
Mouse monoclonal anti-CD3 PercP ef710	eBioscience	Cat: 46-0032-82; RRID: AB_1834427
Mouse monoclonal anti-CD4 BV786	BD Bioscience	Cat: 563727; RRID: AB_2728707
Mouse monoclonal anti-CD8 BV650	BD Pharmigen	Cat: 563234; RRID: AB_2738084
Mouse monoclonal anti-CD44 ef450	eBioscience	Cat: 48-0441-82; RRID: AB_1272246
Mouse monoclonal anti-CD62L BV605	BD Pharmigen	Cat: 56-3252; RRID: AB_2738098
Mouse monoclonal anti-CD11b APC-Cy7	BD Bioscience	Cat: 557657; RRID: AB_396772
Mouse monoclonal anti-CD19 FITC	BD Pharmigen	Cat: 553785; RRID: AB_395049
Mouse monoclonal anti-F4/80 Af647	BD Bioscience	Cat: 565853; RRID: AB_2744474
Rabbit polyclonal anti-MCT1	NovusBio	Cat: NBP1-59656; RRID: AB_11033244
Mouse monoclonal Perforin PE	BioLegend	Cat: 154306; RRID: AB_2721639
Mouse monoclonal IL-17 APC-Cy7	BD Bioscience	Cat: 560821; RRID: AB_2034016
Mouse monoclonal IFNγ Af647	BD Bioscience	Cat: 557735; RRID: AB_396843
Mouse monoclonal Tbet BV421	BD Bioscience	Cat: 563318; RRID: AB_2687543
Mouse monoclonal GATA3 BV711	BD Bioscience	Cat: 565449; RRID: AB_2739242
Mouse monoclonal RORγT PE-CF594	BD Bioscience	Cat: 562684; RRID: AB_2651150
Mouse monoclonal anti-CD8 BUV805	BD Bioscience	Cat: 612898; RRID: AB_2870186
Rabbit polyclonal anti-MCT2	NovusBio	Cat: NBP1-87846; RRID: AB_11022074
Mouse monoclonal anti-MCT4	Santa Cruz Biotechnology	Cat: sc-376140; RRID: AB_10992036
Mouse monoclonal anti-ß-actin	Sigma-Aldrich	Cat: A5441; RRID: AB_476744
Purified anti-mouse CD3 antibody	Biolegend	Cat: 102102; RRDI:AB_312659
Purified anti-mouse CD28 antibody	Biolegend	Cat: 102102; RRID:AB_312867
Rabbit policlonal anti-UCP1	Abcam	Cat: ab10983; RRID: AB_2241462
Peroxidase AffiniPure F(ab')₂ Fragment Goat Anti-Rabbit IgG (H+L)	Jackson ImmunoResearch	Cat: 111-036-045; RRID: AB_2337943
Peroxidase AffiniPure F(ab')₂ Fragment Goat Anti-Mouse IgG (H+L)	Jackson ImmunoResearch	Cat: 115-036-062; RRID: AB_2307346

**Chemicals, peptides, and recombinant proteins**		

LIVE/DEAD Fixable Violet Dead Cell Stain Kit	Thermo fisher	Cat: L34963
RPMI medium	Euroclone	Cat: #ECB2000
Human recombinant IL-2	Peprotech	Cat: #GMP200-02
MitoTracker Green	Invitrogen	Cat: M7514
Mitosox	Invitrogen	Cat: M36008
6-NBDG	Invitrogen	Cat: N23106
AZD3965	Cayman, Vinci Biochem	Cat: CAY-19912-5
Brefeldin A	BD Biosciences	Cat: 555028
CFSE	Merck (Sigma-Aldrich)	Cat: #21888
PMA	Merck (Sigma-Aldrich)	Cat: #P1585
Ionomycin	Invitrogen	Cat: #I24222
DMEM	Merck (Sigma-Aldrich)	Cat: D6429
Penicillin-Streptomycin	Merck (Sigma-Aldrich)	Cat: P0781
Fetal Bovine Serum	Merck (Sigma-Aldrich)	Cat: F7524
3-isobutyl-1-methylxanthine (IBMX)	Merck (Sigma-Aldrich)	Cat: I5869
Dexamethasone	Merck (Sigma-Aldrich)	Cat: D1756
Insulin	Merck (Sigma-Aldrich)	Cat: I6634
Protease inhibitors	Cell Signaling	Cat: #5872S
cOmplete™, Mini, EDTA-free Protease Inhibitor Cocktail	Roche Diagnostics	Cat: 04693159001
PhosSTOP™	Roche Diagnostics	Cat: 04906845001
T-PER buffer	Thermo fisher	Cat: 78510
M-PER buffer	Thermo fisher	Cat: 78501
Novex Sharp Protein Standard	Invitrogen	Cat: LC5801
Amersham™ ECL™ Rainbow™ Marker - Full range	Merck (Sigma-Aldrich)	Cat: GERPN800E
Collagenase NB4 standard grade	Nordmark; Fisher Scientific	Cat: 11427503
10X RBC Lysis Buffer	EBioscience	Cat: 00-4300-54
Formaldehyde solution 37%	Sigma-Aldrich	Cat: 8187081000

**Critical commercial assay**		

EasySep™ Mouse CD8^+^ T Cell Isolation Kit	Stem Cell Technology	Cat: #19853
EasySep™ Mouse CD4^+^ T Cell Isolation Kit	Stem Cell Technology	Cat: #19852
Cholesterol CP kit	ABX Pentra, HORIBA Medical	Cat: A11A01634
Triglyceride CP kit	ABX Pentra, HORIBA Medical	Cat: A11A01640
Maxima First Strand cDNA Synthesis Kit for RT-qPCR	Thermo fisher	Cat: K1642
Maxima SYBR Green/Fluorescein qPCR Master Mix (2X)	Thermo fisher	Cat: K0242
RNeasy Mini Kit (50)	Qiagen	Cat: 74104
TNFα ELISA kit	R&D System	Cat: MHSTA50
Fixation/permeabilization kit	BD Bioscience	Cat: #555028
Clarity Western ECL chemiluminescent substrate	Bio-Rad	Cat: 1705061
Maxima First Strand cDNA synthesis kit	Thermo fisher	Cat: K1641
Seahorse XF Real-Time ATP Rate Assay Kit	Agilent	Cat: 103592-100
Seahorse XF Glycolytic Rate Assay Kit	Agilent	Cat: 103344-100

**Deposited data**		

Metabolomics studies	This paper	https://figshare.com/s/735c0ac5f809f3ae75bd
Proteomics data	This paper	https://doi.org/10.6084/m9.figshare.19709128.v1

**Experimental models: Cell lines**		

NIH-3T3-L1	ATCC	CL-173

**Experimental models: Organisms/strains**		

Mouse: Slc16a1^flox/flox^	Sonveaux Pierre	N/A
Mouse: CD4^cre+^	Marelli-Berg Federica	N/A

**Oligonucleotides**		

Primers: Cebpα Fw: GAACAGCTGAGCCGTGAACT Rev: TAGAGATCCAGCGACCCGAA	Metabion	https://wop.metabion.com/wop/
Primers: Cebpδ Fw: GAACCCGCGGCCTTCTAC Rev: GAAGAGTTCGTCGTGGCACA	Metabion	https://wop.metabion.com/wop/
Primers: Cidea Fw: CACGCATTTCATGATCTTGG Rev: CCTGTATAGGTCGAAGGTGA	Metabion	https://wop.metabion.com/wop/
Primers: Glut4 Fw: GCTCTGACGATGGGGAACC Rev: GCCACGTTGCATTGTAGCTC	Metabion	https://wop.metabion.com/wop/
Primers: Lep Fw: CAAGCAGTGCCTATCCAGA Rev: AAGCCCAGGAATGAAGTCCA	Metabion	https://wop.metabion.com/wop/
Primers: Lpl Fw: TCGGGCCCAGCAACATTATC Rev: TGGTCAGACTTCCTGCTACG	Metabion	https://wop.metabion.com/wop/
Primers: Pparδ Fw: TCTCCCAGAATTCCTCCCCT Rev: GAGCTTCATGCGGATTGTCC	Metabion	https://wop.metabion.com/wop/
Primers: Pparγ Fw: TGTGAGACCAACAGCCTGAC Rev: AAGTTGGTGGGCCAGAATGG	Metabion	https://wop.metabion.com/wop/
Primers: Ppargc1α Fw: CATTTGATGCACTGACAGATGGA Rev: GTCAGGCATGGAGGAAGGAC	Metabion	https://wop.metabion.com/wop/
Primers: Rpl13a Fw:GCGCCTCAAGTGGTGTTGGAT Rev: GAGCAGCAGGGACCACCAT	Metabion	https://wop.metabion.com/wop/
Primers: Slc16a1 Fw: TGTAGGTGCAGCAGCCAAG Rev: TTGAAAGCAAGCCCAAGACC	Metabion	https://wop.metabion.com/wop/
Primers: Tfam Fw: CGGGCCATCATTCGTCG Rev: AGACAAGACTGATAGACGAGGG	Metabion	https://wop.metabion.com/wop/
Primers: Ucp1 Fw: GAGCTGGTAACATATGACCTC Rev: GAGCTGACAGTAAATGGCA	Metabion	https://wop.metabion.com/wop/
Primers: Slc16a1^f/^Fw: GGCATGCCC ATACACACATAAAA Rev: CTGTTTAATCTTGCCGGACATGGTG	Metabion	https://wop.metabion.com/wop/
Primers: Cre Fw: GCGGTCTGGCAGTAAAAACTATC Rev: GTGAAACAGCATTGCTGTCACTT	Metabion	https://wop.metabion.com/wop/
Primers: Cre positive Fw: CTAGGCCACAGAATTGAAAGATCT Rev: GTAGGTGGAAATTCTAGCATCATCC	Metabion	https://wop.metabion.com/wop/

**Software and algorithms**		

Novoexpress	Agilent	https://www.agilent.com/en/product/research-flow-cytometry/flow-cytometry-software/novocyte-novoexpress-software-1320805
QIAGEN's Ingenuity® Pathway Analysis	QIAGEN	www.qiagen.com/ingenuity
Morpheus		https://software.broadinstitute.org/morpheus/(accessed January 22, 2019)
ClustVis		https://biit.cs.ut.ee/clustvis/
GraphPad Prism 8	GraphPad	https://www.graphpad.com/scientific-software/prism
Adobe Photoshop	Adobe	https://www.adobe.com/it/products/photoshop
Proteome Discoverer^TM^ software (version 2.2)	Thermo Fisher	https://www.thermofisher.com/it/en/home/industrial/mass-spectrometry/liquid-chromatography-mass-spectrometry-lc-ms/lc-ms-software/multi-omics-data-analysis/proteome-discoverer-software.html
MultiQuant™ software (version 3.0.3)	AB Sciex, Framingham, MA	https://sciex.com/products/software/multiquant-software

**Other**		

Standard fat diet (SFD)	Research diet INC	Cat: D12450H
High fat diet (HFD)	Research diet INC	Cat: D12451
Chow diet	ssniff Spezialdiäten GmbH	Cat: V1534-300
Novex NuPAGE 4-12% Bis-Tris Mini Gels	Invitrogen	Cat: NP0322BOX
Bovine Serum Albumin	AppliChem GmbH	Cat: A1391
Zip Tip with 0.6 μL C_18_ resin	Millipore	Cat: ZTC18S008
Acclaim™ PepMap™ 100C18 HPLC Columns	Thermo Scientific	Cat: 164199
EASY-Spray™ HPLC Columns	Thermo Scientific	Cat: ES900

### Resource availability

#### Lead contact

Further information and reasonable requests for resources and reagents should be directed to the lead contact: Massimiliano.ruscica@unimi.it.

#### Materials availability

This study did not generate any unique materials.

### Experimental model and subject details

#### Mice and treatment

Mice carrying floxed allels of *Slc16a1* (termed *Slc16a1*^*f/f*^) on the C57BL/6 genetic background were bred with CD4^cre+^ mice (kindly given by Prof. Marelli-Berg) for specific deletion of *Slc16a1* in both CD4^+^ and CD8^+^ T lymphocytes. Mice developed normally, were fertile, with behavioural and functional parameters within normal range. B6.Slc16a1^fl/fl^ mice were generated using homologous recombination in mouse embryonic stem (ES) cells at the Université catholique de Louvain in collaboration with Genoway (ethical authorization #UCL/MD/2010/11). Briefly, a 10.5 kB fragment of the mouse Slc16a1 locus was retrieved from an artificial bacterial chromosome (RP23-208N5, Chori) and modified using a gap repair strategy in the pL253 vector with the λ-prophage-based system. We floxed exons 2 and 3, where the lactate and proton recognition sites are located. The vector was then amplified, purified, linearized, and used for C57BL/6 ES cell transfection by electroporation at Genoway. After selection, ES cells were injected in C57BL/6J blastocysts to obtain B6.Slc16a1^fl/fl^ chimeric mice that were regularly genotyped.

Animals carrying the CD4^cre^ allele were used as knockout (KO) (termed *Slc16a1*^*f/f*^*Tcell*^*cre*^) and the Cre-negative littermates were used as wildtype (WT) (*Slc16a1*^*f/f*^). For genotyping of mice, DNA derived from ear biopsies was amplified by PCR using primers detecting the floxed alleles and the presence of the Cre. Seven-week-old mice were housed four per cages and kept in a temperature-controlled environment (20 ± 2°C, 50 ± 5% relative humidity) with a 12-h light/dark cycle in an air-conditioned room and free access to food and water. Starting from 8 weeks of age, male littermates *Slc16a1*^*f/f*^ and *Slc16a1*^*f/f*^*Tcell*^*cre*^ mice were randomized in two groups, one fed a standard fat diet (SFD, 10% Kcal from fat), and the other one fed a high fat diet (HFD, 45% Kcal from fat). Food intake and weight gain were measured weakly. After 20 weeks of diet, mice were sacrificed by isoflurane (2%) inhalation and cervical dislocation, and epididymal white adipose tissue, subcutaneous adipose tissues (epiWAT and SCAT, respectively), spleen, mesenteric lymph node, thymus and liver were collected and weighted. Blood was drawn by cardiac puncture at sacrifice. All animal procedures were performed in accordance with the guidelines from directive 2010/63/EU of the European Parliament on the protection of animals used for scientific purposes and were approved by the Ethical Committee (Authorization 780/2016 to MR).

### Method details

#### Blood biochemistry measurements, glucose, and insulin tolerance tests

Plasma was separated by centrifugation at 8,000 rcf for 10 min. Cholesterol and triglycerides were measured using standard enzymatic techniques (Cholestrol CP kit, ABX Pentra, HORIBA Medical; Triglyceride CP kit, ABX Pentra, HORIBA Medical). *Glucose tolerance test (GTT)*: Mice were fasted overnight (12–16 h), weighed and blood glucose was measured by snipping the tail and using a glucometer (One-Touch Ultra). Glucose solution (2 g/kg body weight) was injected intraperitoneally and glycemia measured after 20, 40, 60, 120 min. *Insulin Tolerance Test (ITT)*: Mice were fasted for 4 h, weighed and blood glucose was measured as previously described. Insulin solution (0.2 IU/kg body weight) was injected in the intraperitoneal cavity and mice were bled as described above after 20, 40, 60, 120 min. The area under the curve (AUC) was calculated for glucose clearance following GTT or ITT.

#### RNA isolation and quantitative PCR

Real-Time PCR analysis was performed on a CFX Connect Real-Time System (Biorad) using the iTaq Universal SYBR Green supermix (Thermo Fisher). qPCR conditions were: 3 min at 95°C, 40 cycles of 10 s at 95°C, 30 s at 60°C. The relative expression of messenger RNA (mRNA) was calculated with the comparative CT (^ΔΔ^CT) method using Rpl13a as endogenous control gene. The relative mRNA levels were expressed as fold change. Melting curve analysis was performed to verify the specificity of the PCR products.

#### Western blot analysis

Depending on the experiments, proteins from tissues or cells have been extracted by TPER or MPER buffers, respectively (Thermo Fisher), containing a cocktail of protease and phosphatase inhibitors (Roche Diagnostics). Ten μg of proteins and a molecular mass marker (Novex Sharp Protein Standard, Invitrogen^TM^; Life Technologies) were separated on 4-12% sodium dodecylsulfate-polyacrylamide gel (SDS-PAGE; Novex NuPAGE 4-12% Bis-Tris Mini Gels, Invitrogen; Life Technologies) under denaturing and reducing conditions. Proteins were then transferred to a nitrocellulose membrane by using the iBlotTM Gel Transfer Device (Invitrogen; Life Technologies). The membranes were washed with Tris-Buffered Saline-Tween 20 (TBS-T) and non-specific binding sites were blocked in TBS-T containing 5% BSA (SIGMA-Aldrich) for 90 min at room temperature. The blots were incubated overnight at 4°C with a diluted solution (5% BSA or non-fat dried milk) of the human primary antibodies (Rabbit polyclonal anti-MCT2, NovusBio NBP1-87846; Mouse monoclonal anti-MCT4, Santa Cruz Biotechnology sc-376140; mouse monoclonal anti-βactin, Sigma-Aldrich A5441; rabbit policlonal UCP1, abcam ab10983). Membranes were washed with TBS-T and then exposed for 90 min at RT to a diluted solution (5% non-fat dried milk) of the secondary antibodies (anti-mouse and anti-rabbit peroxidase-conjugated secondary antibodies (Jackson ImmunoResearch). Immunoreactive bands were detected by exposing the membranes to Clarity Western ECL chemiluminescent substrates (Bio-Rad Laboratories) for 5 min and images were acquired with a ChemiDoc XRS System (Bio-Rad Laboratories). Densitometric readings were evaluated using the ImageLab software.

#### ELISA—Enzyme-linked immunosorbent assay

TNF-α concentrations in serum and epiWAT of *Slc16a1*^*f/f*^ and *Slc16a1*^*f/f*^*Tcell*^*cre*^ mice were measured by a commercial ELISA kit. Undiluted serum samples and diluted (1:2) epiWAT samples were incubated onto a microplate pre-coated with a monoclonal mouse-TNFα-specific antibody. Sample concentrations were obtained by a four-parameter logistic curve-fit, with a minimum detectable TNF-α concentration of 0.081 pg/mL.

#### Samples preparation for immunophenotyping by flow cytometry

After the addition of collagenase (200 mg/mL final concentration, NB4 standard grade) and CaCl_2_ (5 mM final concentration) samples were incubated at 37°C for 40 min under agitation. Samples were then topped up with MACS buffer (PBS, 2% FCS, 2 mM EDTA) and filtered. After the lysis of red blood cells for 5 min on ice, samples were washed, spun and resuspended in 50 μL of antibodies mix. Mesenteric lymph nodes and spleen were processed on a 70μm cell strainer and red blood cells eliminated with red blood cell lysis buffer for 5 min on ice. Cells were then washed and resuspended in antibodies mix (30 min at 4°C). Samples were acquired with Novocyte3000 (ACEA Bioscience).

#### Histology

Epididymal white adipose tissue (epiWAT) and subcutaneous adipose tissue (SCAT) were collected and left overnight in 4% buffer formalin at 4°C. The quantification of the average area of adipocyte was assessed as follows: 5μm sections were stained with haematoxylin and eosin and acquired using the axiovision Zeiss software (10x magnification), followed by the quantification of adipocytes area using Adobe Photoshop software after the manual selection of an area with intact adipocytes (Bonacina et al., 2019).

#### *In vitro* T cell activation, cytokines quantification and co-culture experiments

Primary lymphocytes were isolated from the lymph nodes and spleen of *Slc16a1*^*f/f*^ and *Slc16a1*^*f/f*^*Tcell*^*cre*^ mice and a uniform cell suspension was prepared by mashing the lymph nodes through a 70 μm cell strainer with 1mL syringe plunger with PBS/2% FBS/2 μM EDTA PBS (MACS buffer). Cells were spun at 500 g for 5 min, lysed with red blood lysis buffer for 5 min at 4°C – when depletion of red blood cells was required – suspended in MACS buffer and counted. CD8^+^ T cells and CD4^+^ T cells purification were performed with commercial kits (EasySep™) when indicated. For proliferation analysis, lymphocytes suspension from murine lymphoid organs were stained with 5 μM of CFSE (Merck) for 10 min at RT in dark, diluted 10 times in PBS/FBS 2%/2mM EDTA, washed 3 times and resuspended in warm media. 0.2 × 10^6^ cells were plated with 200 μL of complete RPMI medium (R10; RPMI plus 10% FBS, glutamine, HEPES, MeOH, sodium pyruvate and antibiotics) in the presence of IL-2 (25 U/mL) in 96-well plate (U-bottom wells), previously coated with 0.5 μg/mL anti-CD3 and 2.5 μg/mL CD28. In the experiment involving the use of MCT1 inhibitor, AZD3965 was added at 25nM were indicated. After 4 days at 37°C with 5% of CO_2_, proliferation was assessed as CFSE dilution by flow cytometry. For cytokine production, 96 h after the culture, cells were pulsed with 0.1 μg/mL PMA and 1 μg/mL ionomycin for 4 h at 37°C with 5% of CO_2_ in the presence of Brefeldin A (1:1000). Cytokine analysis was performed by flow cytometry following the instructions from the fixation/permeabilization kit. MCT1 mean fluorescent intensity (MFI) was acquired by flow cytometry after overnight stimulation with anti-CD3 and anti-CD28 as described above. Superficial staining, with antibodies against CD4, CD8, CD62l and CD44 proteins and primary antibody for MCT1, was performed for 30 min at 37°C, followed by secondary antibody staining for MCT1, 30 min at 37°C. 6-NBDG was assessed by flow cytometry after 2 days of stimulation and 30 min of incubation with the 6-NBDG dye in PBS followed by superficial staining with anti-CD8 antibody. Mitotracker green and Mitosox staining was done at 96 h of stimulation in order to evaluate mitochondrial mass and ROS production. Mitotracker was added for the staining together with anti-CD8 antibody for 30 min at RT, while Mitosox dye was incubated with cells after superficial staining and leaved 10 min at 37°C. Relative to co-culture experiments, first of all, NIH-3T3 preadipocytes have been differentiated in mature adipocytes as follows: cells were cultured for 2 days to confluence (day 0), and adipogenic differentiation was induced by treatment with DMEM containing 10% FBS, 2 μg mL^−1^ insulin, 1 μM dexamethasone, and 0.5 mM 3-isobutyl-1-methylxanthine (IBMX) for 2 days. After the induction of differentiation, the medium was changed to differentiation-maintenance medium containing 10% FBS and insulin and was replaced every 2 days. Second, activated CD8^+^ T cells either KO or WT for MCT1 have been co-cultured by NIH-3T3 adipocytes 3 days before the end of the differentiation protocol. At day ten, cells were harvested, and mRNA extracted for the evaluation of gene expression.

#### Proteomics analysis

Proteomics analysis was performed on purified CD4^+^ and CD8^+^ T lymphocytes subsets (2 × 10 ^6^ cells per subset) from *Slc16a1*^*f/f*^ and *Slc16a1*^*f/f*^*Tcell*^*cre*^ mice (n = 6, respectively) in resting conditions or after 96-h of stimulation. Samples were pooled together for each condition and lysed in the presence of Urea 8.0 M in 0.1 M Tris-HCl buffer (pH 8.5) and protease inhibitors (Cell Signaling) at a ratio of 1:100. They were then homogenized and incubated for 30 min at 4°C. Subsequently, they were centrifuged at 10,000×g for 20 min at 4°C and the supernatant containing the protein extract was collected. The protein content was measured with Bradford Protein Assay.

Using a vacuum concentrator (45°C, 45 min), 10 μg of samples were then dried and were resuspended in 50mM ammonium bicarbonate buffer to a final volume of 60 μL. The pH was checked to be 8.5. Proteins were reduced in 5.0 mM DTT for 30 min at 55°C. Protein alkylation was then performed at RT, with 15mM iodoacetamide, for 30 min in the dark. For protein digestion, trypsin was added at an enzyme-to-protein ratio 1:20 for 16 h at 37°C. The digested samples were acidified by addition of trifluoroacetic acid 50% (w/w). Final protein concentration was 0.13 μg/μL. The proteolytic peptide mixtures were purified by C_18_ resine pipette tips and analysed in triplicate by nLC-MS/MS.

#### LC-MS/MS analysis

Samples were analysed at UNITECH OMICs (University of Milan, Italy) using a Dionex Ultimate 3000 nano-LC system (Sunnyvale CA) connected to an orbitrap Fusion™ Tribrid™ Mass Spectrometer (Thermo Fisher) equipped with a nanoelectrospray ion source operating in positive ion mode. The peptide mixtures were pre-concentrated onto an Acclaim PepMap C18 column and separated at 35°C on an EASY-Spray PepMap RSLC C18 column. Peptides were eluted with gradient runs from 96% buffer A (0.1% aqueous formic acid) to 40% buffer B (0.1% aqueous formic acid /acetonitrile (2:8)). Run total length: 110 min. Flow rate: 300 nL min^−1^. MS spectra were collected over an m/z range of 375 – 1500 Da at resolution 120,000 in the data dependent mode, cycle time 3 s between master scans. Fragmentation was induced by higher energy collisional dissociation (HCD) with collision energy set at 35 eV.

#### Metabolomics

Metabolomics data were obtained by liquid chromatography coupled to tandem mass spectrometry. We used an API-3500 triple quadrupole mass spectrometer (AB Sciex, Framingham, MA) coupled with an ExionLC™ AC System (AB Sciex, Framingham, MA). Cells were resuspended in 250μL of ice-cold methanol/water 50:50 containing internal standards. Lysates were spun at 20,000g for 5 min at 4°C and supernatants were then transferred to deep-well 96 plate. Samples were then dried under N2 flow at 40°C. Samples were then resuspended in 100μL of methanol for subsequent analysis.

Quantification of energy metabolites was performed by using a cyano-phase LUNA column (50 mm × 4.6 mm, 5μm; Phenomenex, Torrance, CA, USA) by a 5 min run in negative ion mode with two separated runs:

Protocol A was used to analyse lactate, malate, αKetoglutarate, phosphoenolpyruvate (PEP), dihydroxyacetone-P/glyceraldehyde-3P (DHAP/GAP), erytrose-4P (E4P), dTMP, dAMP, dIMP, dCTP, ITP and GTP. The mobile phase A was: water and phase B was: 5mM ammonium acetate in MeOH and the gradient was 90% B for all the analysis with a flow rate of 500 μL/min.

Protocol B was used to analyse 3′, 5′-Cyclic GMP, Acetyl-CoA, ADP, AMP, ATP, cAMP, Citrate, CMP, CoA, CTP, dADP, dATP, dCDP, dCMP, dGDP, dGMP, dGTP, dITP, dTTP, dUMP, dUTP, FAD, Fructose bis-P, Fumarate, GDP, Glucose, Glucose-6P, GMP, IMP, Iso-citrate, malonyl-CoA, NAD+, NADH, NADP+, NADPH, oxaloacetate, pyruvate, ribose-xylulose-ribulose-5P (R-X-Ru-5P), succinate, succinyl-CoA, UDP, UMP and UTP. The mobile phase A was: water and phase B was: 5mM ammonium acetate in MeOH and the gradient 50% B for all the analysis with a flow rate of 500 μL/min.

Carnitine quantification was performed on acetonitrile/methanol extracts by using a Varian Pursuit XRs Ultra 2.8 Diphenyl column. Samples were analysed by a 3 min run in positive ion mode and the mobile phase was 0.1% formic acid in MeOH. Amino acid and biogenic amine quantification was performed through previous derivatization. Briefly, 20μL out of 100μL of acetonitrile/methanol samples were collected and dried under N2 flow at 40°C. Dried samples were resuspended in 50μL of phenyl-isothiocyanate (PITC), EtOH, pyridine and water 5%:31.5%:31.5%:31.5% and then incubated for 20 min at RT, dried under N2 flow at 40°C for 90 min and finally resuspended in 100μL of 5mM ammonium acetate in MeOH/H_2_O 50:50. Quantification of different amino acids was performed by using a C18 column (Biocrates, Innsbruck, Austria) maintained at 50°C. The mobile phases for positive ion mode analysis were phase A: 0.2% formic acid in water and phase B: 0.2% formic acid in acetonitrile. The gradient was T0: 100%A, T5.5: 5%A, T7: 100%A with a flow rate of 500 μL/min. All metabolites analysed in the described protocols were previously validated by pure standards and internal standards were used to check instrument sensitivity.

#### Seahorse assay

Activated CD8^+^ T cells were resuspended in culture medium (DMEM Medium, pH 7.4 with 10 mM glucose, 1 mM pyruvate, 2 mM glutamine) and plated in a XF24 cell culture microplate (4 × 10^5^ cells per well). The microplate was left 1 h in a CO_2_-deprived incubator (37°C) and then loaded in a Seahorse XFe24 Analyzer (Agilent Technologies, Santa Clara, USA). To distinguish between the fractions of ATP produced from mitochondrial oxidative phosphorylation (OXPHOS) and those produced by glycolysis, oxygen consumption rate (OCR) and extracellular acidification rate (ECAR) were measured at baseline and after subsequent injections of Oligomycin (1.5 μM) and Rotenone plus antimycin A (0.5 μM) (Seahorse ATP Real-Time rate assay). To further shed light on T cell glycolysis Seahorse XF Glycolytic Rate Assay was used. Rates at basal level as well as after injections of Rotenone plus antimycin A (1 μM) and 2-deoxy-D-glucose (50 mM) were recorded.

### Quantification and statistical analysis

#### Proteomic analysis

The raw mass spectrometry files were processed, searched and label free quantified (LFQ) using Proteome Discoverer^TM^ software (version 2.2, Thermo Fisher). Proteins and peptides were identified using the UniProt mouse database (uniprot-mouse.fasta). The parameters were set as follows: enzyme trypsin, peptide≥2, carbamidomethylation of cysteine residues and oxidation were set as fixed modification, N-terminal protein acetylation was selected as variable modification. PSM (peptide-spectrum matches) Xcorr >= 2.2. Master proteins, determined in Proteome Discoverer^TM^ software, were normalized values for high confidence (1% protein-level FDR). To compare proteome of subsets, LFQ values were log2-transformed and the 3 technical replicates per experimental condition grouped. The proteome used for PCA is the entire list of proteins, after imputation of the missing values. Unit variance scaling is applied to rows; SVD with imputation is used to calculate principal components. X and Y axis, of the PCA analysis, show principal component 1 and principal component 2 that explain 80.1 and 8.2% of the total variance, respectively. N = 12 data points.

#### Metabolomic analysis

MultiQuant™ software was used for data analysis and peak review of chromatograms (Audano et al., 2021). Raw areas were normalized by the median of all metabolite areas in the same sample. The data were then transformed by generalized log-transformation and Pareto scaled to correct for heteroscedasticity, reduce the skewness of the data, and reduce mask effects. In detail, obtained values were transformed by generalized log (glog) as follows:

$$g\;\log_{2}\left(x\right)=\log_{2}\frac{x+\sqrt{x^{z}+a^{z}}}{2}$$

where a is a constant with a default value of 1 and x is the sample area for a given metabolites. Then, obtained values underwent Pareto scaling as follows:

$${\bar{X}}_{ij}=\frac{x_{ij}{\bar{x}}_{i}}{\sqrt{S_{i}}}$$

where x_ij_ is the transformed value in the data matrix (i (metabolites), j (samples)) and s_i_ is the standard deviation of transformed metabolite values. Obtained values were considered as relative metabolite levels. Data processing and analysis were performed by MetaboAnalyst 5.0 web tool.

#### Statistical analysis

Results are presented as mean per group ± SEM and analysed according to what reported in figure legends. A p value <0.05 (∗p < 0.05, ∗∗p < 0.01) was considered significant. Group differences were evaluated by non-parametric Kruskal–Wallis one-way analysis and Mann-Whitney test. If a normal distribution was present, parametric tests were used. For multiple comparisons, a one-way ANOVA with a 95% confidence interval was used. Pertaining metabolomic evaluations, differences between groups were assessed by Fisher’s Least Significant Difference test with false discovery rate (FDR) correction; ^∗^FDR < 0.1, ^∗∗^FDR < 0.05. Statistical analysis was performed through Excel and GraphPad-Prism8. For proteomic interpretation, pathways analysis for the data sets were analysed using the use of QIAGEN’s Ingenuity® Pathway Analysis (IPA®, QIAGEN Redwood City, CA). Further analyses for enrichment of functional and biological function were performed using STRING and KEGG database. Hierarchical clustering using person’s correlation was performed by heatmap after z-score normalization using Morpheus and ClustVis (Metsalu and Vilo, 2015) was used to perform principal component analysis (PCA).

## Data Availability

Data pertaining to proteomic and metabolomic analyses are available at the following links: https://figshare.com/s/735c0ac5f809f3ae75bd. https://doi.org/10.6084/m9.figshare.19709128.v1. Any additional information required to reanalyse the data reported in this paper is available from the lead contact upon request. This paper does not report original code.
