# Supplementary material for: Monocarboxylate transporter 1 deficiency impacts CD8+ T lymphocytes proliferation and recruitment to adipose tissue during obesity
Source: iScience. 2022 May 23;25(6):104435. doi: 10.1016/j.isci.2022.104435 (PMC9189020; doi:10.1016/j.isci.2022.104435)
Supplement: Document S1. Figures S1–S6 [file mmc1.pdf]

**iScience, Volume *25***

## Supplemental information

# Monocarboxylate transporter 1 deficiency impacts CD8$^+$ T lymphocytes proliferation and recruitment to adipose tissue during obesity

**C. Macchi, A. Moregola, M.F. Greco, M. Svecla, F. Bonacina, S. Dhup, R.K. Dadhich, M. Audano, P. Sonveaux, C. Mauro, N. Mitro, M. Ruscica, and G.D. Norata**

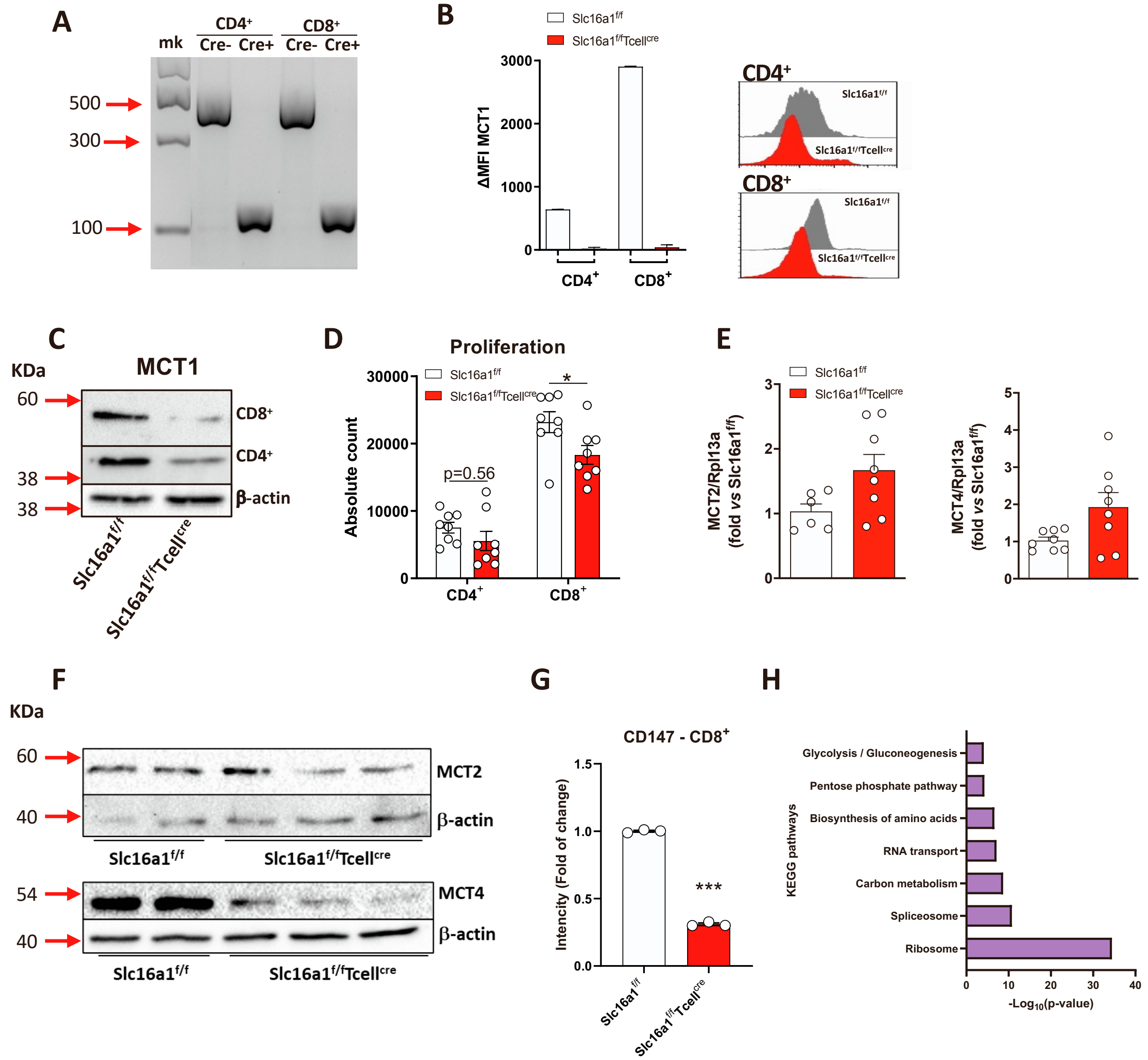


**I**

| | PC1 | PC2 | PC3 | PC4 | PC5 | PC6 | PC7 | PC8 | PC9 | PC10 | PC11 | PC12 |
|---|---|---|---|---|---|---|---|---|---|---|---|---|
| Individual | 0.80 | 0.08 | 0.04 | 0.04 | 0.01 | 0.01 | 0.01 | 0.01 | 0.01 | 0.00 | 0.00 | 0.00 |
| Cumulative | 0.80 | 0.88 | 0.93 | 0.96 | 0.97 | 0.98 | 0.98 | 0.99 | 0.99 | 1.00 | 1.00 | 1.00 |

| | Before processing | After collapsing similar columns (if applied) | After removing rows and columns with NAs | After removing constant rows and optionally columns |
|---|---|---|---|---|
| Rows | 1216 | 1216 | 1216 | 1216 |
| Columns | 12 | 12 | 12 | 12 |

**Figure S1. [Characterization of MCT1-deficient $CD8^+$ T cells], Related to Figure 1.**
A. End-point PCR confirming the deletion of exon 2 and 3 of *Slc16a1* gene in $CD4^+$ or $CD8^+$ T cells isolated from $Slc16a1^{f/f}$ and $Slc16a1^{f/f}Tcell^{cre}$ mice.
B. MCT1 expression presented as MFI in $CD4^+$ and $CD8^+$ T cells isolated from $Slc16a1^{f/f}$ and $Slc16a1^{f/f}Tcell^{cre}$ mice; n=2 per group.

C. Western Blot analysis of MCT1 expression in $CD4^+$ and $CD8^+$ T cells from $Slc16a1^{f/f}$ and $Slc16a1^{f/f}Tcell^{cre}$ mice after in vitro stimulation with anti-CD3, anti-CD28 and IL-2.
D. Proliferation of $CD4^+$ and $CD8^+$ T cells isolated from *Slc16a1*$^{f/f}$ and *Slc16a1*$^{f/f}$*Tcell*$^{cre}$ mice after *in vitro* activation with anti-CD3 and anti-CD28 for 4 days. Data are expressed as absolute count. n=8 per group.
E. Gene espression of MCT2 and MCT4 in $CD8^+$ T cells isolated from $Slc16a1^{f/f}$ and $Slc16a1^{f/f}Tcell^{cre}$ mice after *in vitro* stimulation with anti-CD3, anti-CD28 and IL-2. n=3-4 per group
F. Western Blot analysis of MCT2 and MCT4 in $CD8^+$ T cells isolated from $Slc16a1^{f/f}$ and $Slc16a1^{f/f}Tcell^{cre}$ mice after *in vitro* stimulation with anti-CD3, anti-CD28 and IL-2.
G. CD147 expression in $CD8^+$ T cells isolated from $Slc16a1^{f/f}$ and $Slc16a1^{f/f}Tcell^{cre}$ mice after *in vitro* stimulation with anti-CD3, anti-CD28 and IL-2, as assessed by proteomic analysis. n= 3 per group.
H. Enrichment pathway analysis comparing $CD8^+$ T cells isolated from $Slc16a1^{f/f}$ and $Slc16a1^{f/f}Tcell^{cre}$ mice after *in vitro* activation with anti-CD3 and anti-CD28 for 4 days.
Cut off p-value for protein intensity lower 0.05 and cut off for pathway FDR lower 0.05.
I. PCA proteome was generated with the web tool ClustVis after imputation of the missing values. Unit variance scaling is applied to rows; SVD with imputation is used to calculate principal components. X and Y axis show principal component 1 and principal component 2 that explain 80.1% and 8.2% of the total variance, respectively. n= 12 data points.
In (B, D, F) data are presented as mean ± SEM. Differences between groups have been assessed by unpaired two-sides T-test or by two-way ANOVA or Kruskal-Wallis non-parametric test. ∗∗∗p < 0.001 versus respective control. MCT, Monocarboxylate transporter; MFI, media fluorescence intensity; Slc16a1, solute carrier family 16 member 1.

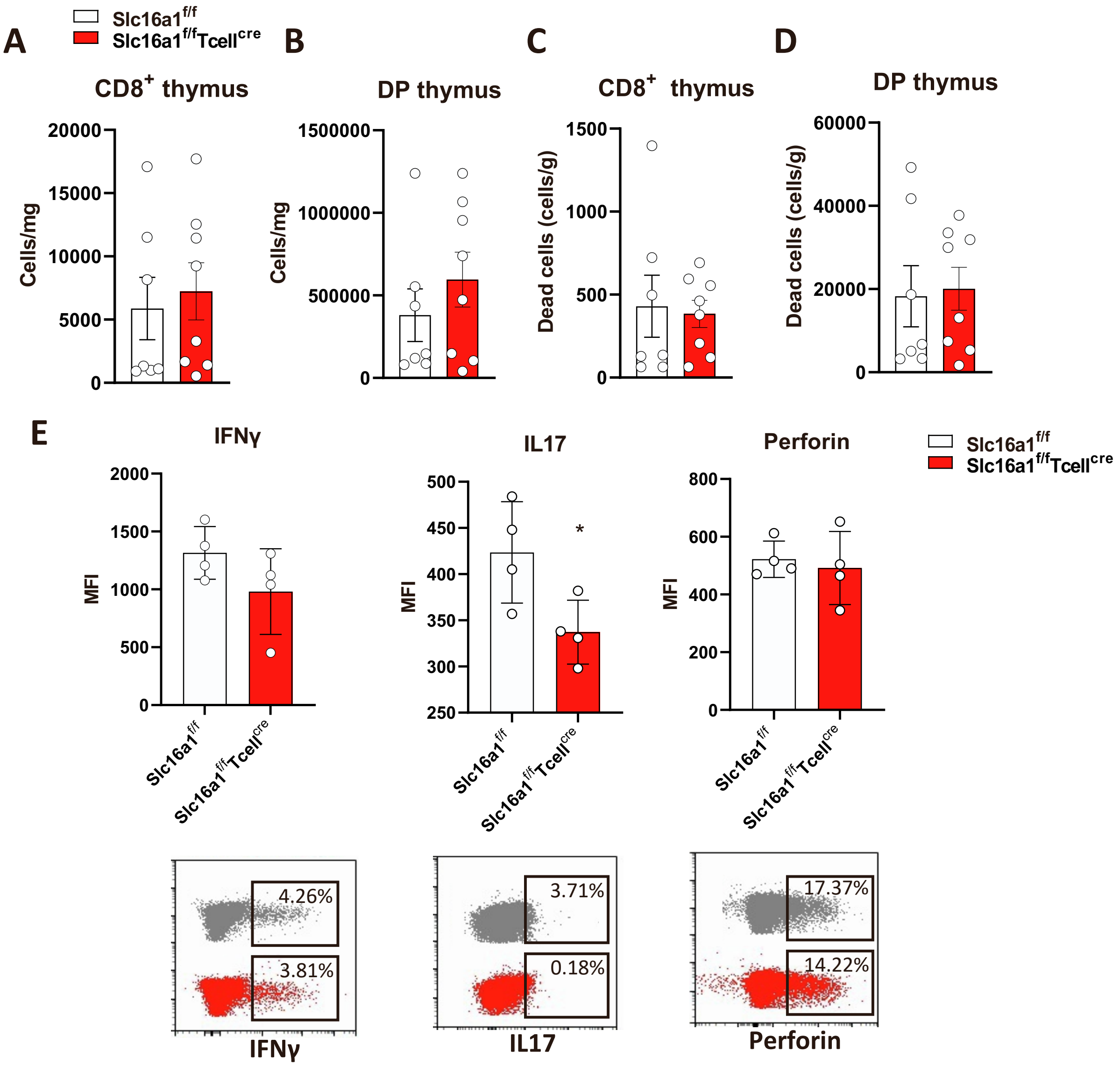


**Figure S2. [Characterization of MCT1-deficient CD8$^+$ T cells], Related to Figure 1.**
A-B. Number of total thymic CD8$^+$ Tcell (A) and number of total thymic CD4$^+$CD8$^+$ T cells (B) (DP, double positive). Positive for aqua viability staining. n=7-8 per group.
C-D. Number of total thymic CD8$^+$ dead cells (C) and CD4$^+$CD8$^+$ T dead cells (D) (DP, double positive), positive for live/dead viability staining; n=7-8 per group.
E. Cytokines production, presented as MFI, in CD8$^+$ T cells isolated from *Slc16a1$^{f/f}$* and *Slc16a1$^{f/f}$Tcell$^{cre}$* mice after *in vitro* activation with anti-CD3 and anti-CD28; n= 4 per group.
Cut off p-value for protein intensity lower 0.05.
In (A-E) data are presented as mean ± SEM. Differences between groups have been assessed by unpaired two-sides T-test or Kruskal-Wallis non-parametric test. $*p < 0.05$ versus respective control. MFI, media fluorescence intensity; IFNγ, Interferon γ; IL, Interleukin; Slc16a1, solute carrier family 16 member 1; MFI (median fluorescent intensity)

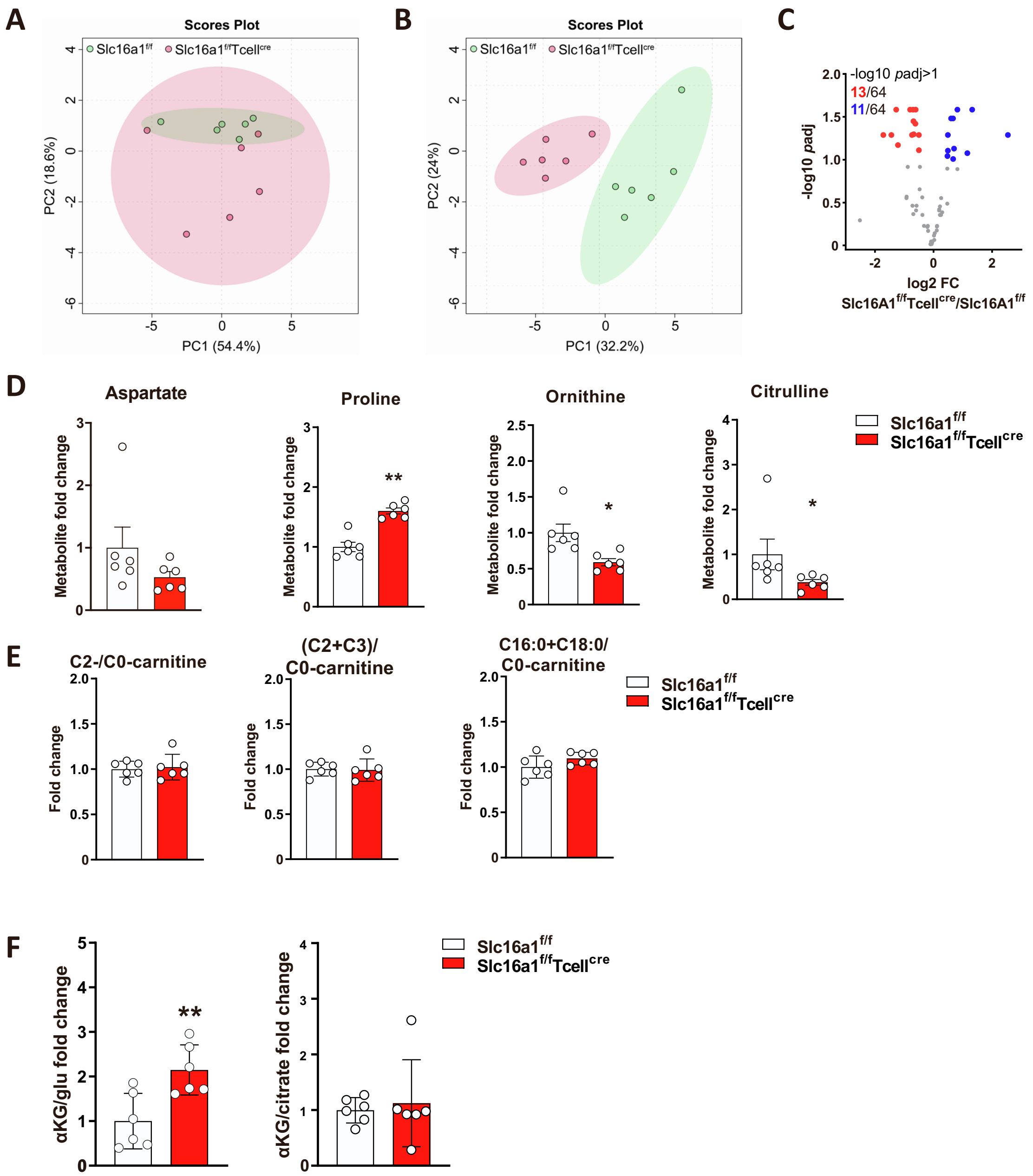


**Figure S3. [Energy metabolism of CD8$^+$ T cells lacking MCT1], Related to Figure 3.**
A-B. Principal component analysis (PCA) of inactivated (A) and 4 days activated (B) CD8$^+$ T cells isolated from *Slc16a1$^{f/f}$* and *Slc16a1$^{f/f}$Tcell$^{cre}$* mice. n=6.
C. Volcano plot representing singificantly downregulated (red dots), upregulated (blue dots) and unaffected (gray dots) metabolites in 4 days activated *Slc16a1$^{f/f}$Tcell$^{cre}$* CD8$^+$ T cells compared to *Slc16a1$^{f/f}$* control. Metabolites with a FDR < 0.1 were considered statistically significant.
D. Relative levels of indicated metabolites from LC-MS/MS targeted metabolomics of CD8$^+$ T cells isolated from *Slc16a1$^{f/f}$* and *Slc16a1$^{f/f}$Tcell$^{cre}$* mice after *in vitro* activation with anti-CD3 and anti-CD28 for 4 days.
E. Ratio of indicated metabolites in CD8$^+$ T cells isolated from *Slc16a1$^{f/f}$* and *Slc16a1$^{f/f}$Tcell$^{cre}$* mice after *in vitro* activation with anti-CD3 and anti-CD28 for 4 days. (n=6 per group)
F. Ratio of indicated metabolites in CD8$^+$ T cells isolated from *Slc16a1$^{f/f}$* and *Slc16a1$^{f/f}$Tcell$^{cre}$* mice after *in vitro* activation with anti-CD3 and anti-CD28 for 4 days. (n= 6 per group).

Data are presented as mean ± SEM and differences between groups have been assessed by Fisher's LSD test with FDR correction. *FDR < 0.1, **FDR < 0.05 *versus* respective control.
LC-MS/MS, Liquid Chromatography with tandem mass spectrometry; LSD, Least Significant Difference; Slc16a1, solute carrier family 16 member 1.

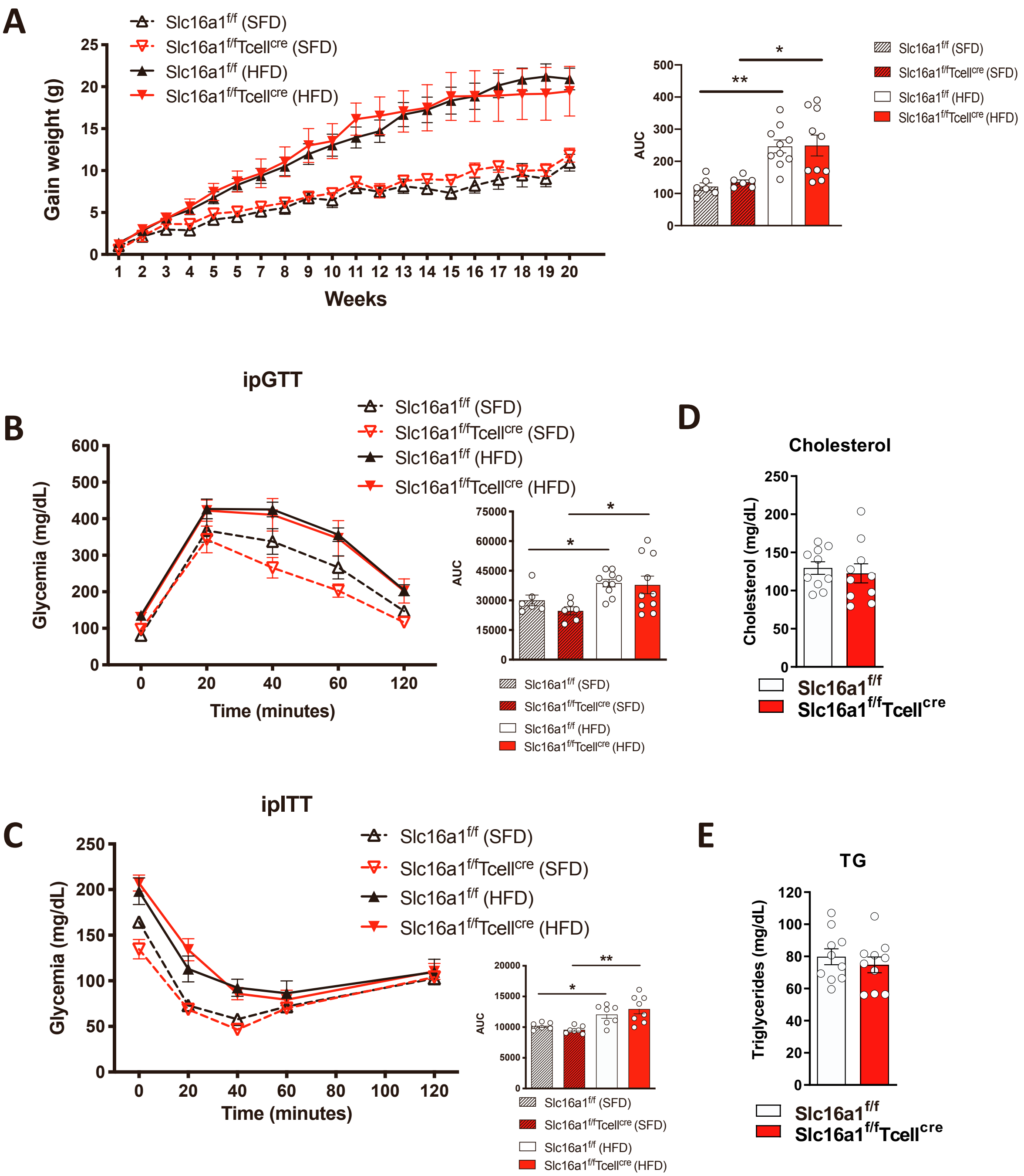


**Figure S4. [Gluco-metabolic profile of *Slc16a1^f/f^* and *Slc16a1^f/f^Tcell^cre^* mice], Related to Figure 4.**
A. Weight gain of *Slc16a1$^{f/f}$* and *Slc16a1$^{f/f}$Tcell$^{cre}$* mice fed a HFD (n=10 per group) or a SFD (n=6 per group) for 20 weeks.
B, C. ipGTT (B) and ipITT (C) performed on *Slc16a1$^{f/f}$* and *Slc16a1$^{f/f}$Tcell$^{cre}$* mice fed a HFD for 20 weeks (ipGTT, n=10 per group; ipITT, n=8-9 per group) or a SFD for 20 weeks (ipGTT, n=6 per group; ipITT, n=6-7 per group).
D, E. Quantification of plasma cholesterol (D) and triglycerides (E) of *Slc16a1$^{f/f}$* and *Slc16a1$^{f/f}$Tcell$^{cre}$* mice fed a HFD for 20 weeks; n=10 per group.
In (A-E) data are presented as mean ± SEM. Differences between groups have been assessed by unpaired two-sides T-test or by two-way ANOVA. *$p < 0.05$, **$p < 0.01$ *versus* respective control.
AUC, area under curve; HFD, high fat diet; ipGTT, intraperitoneal glucose tolerance test; ipITT, intraperitoneal insulin tolerance test; Slc16a1, solute carrier family 16 member 1; SFD, standard fat diet; TG, triglycerides.

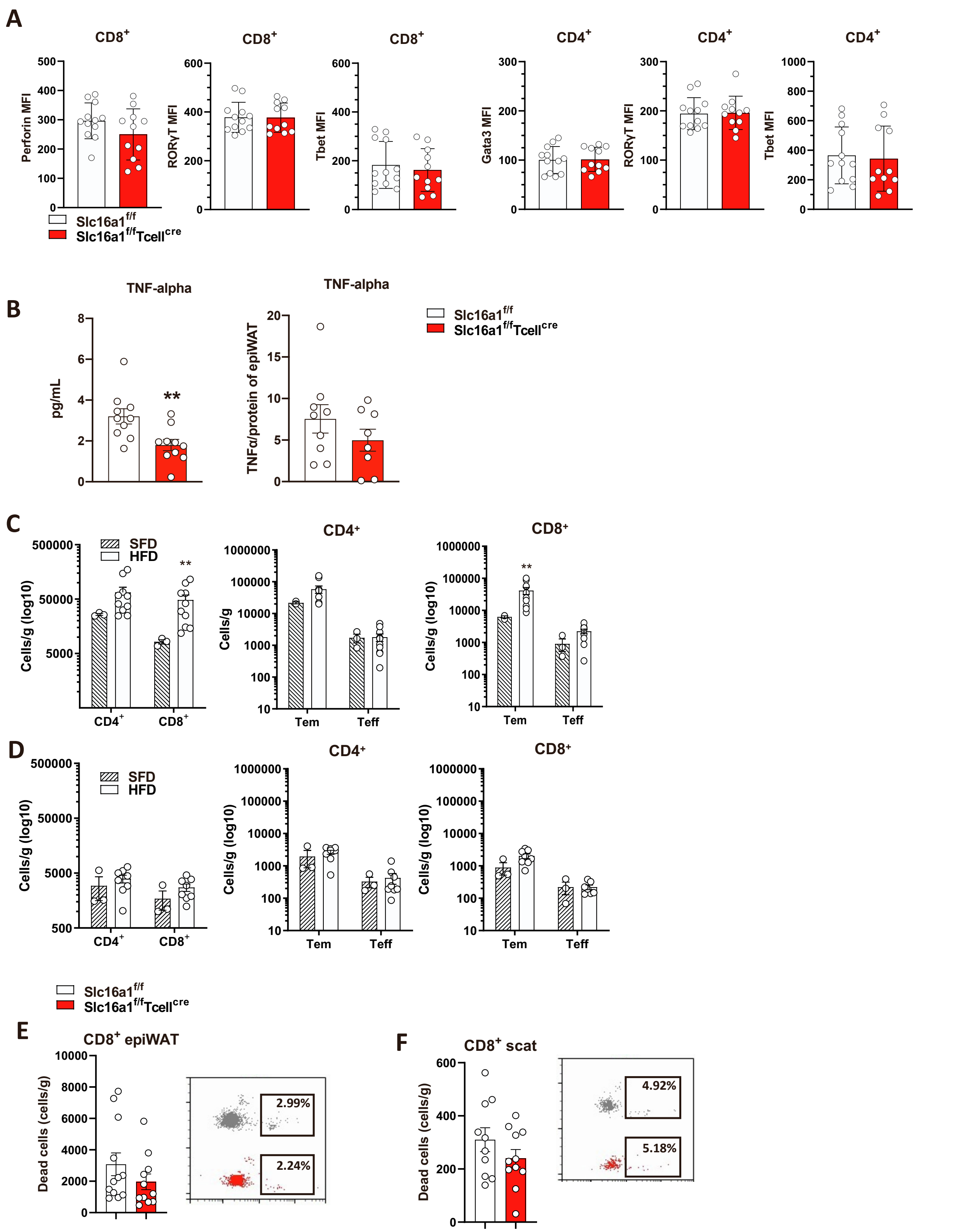


**Figure S5. [Immuno-metabolic signature of obese mice selectively lacking MCT1 in T cells], Related to Figure 6.**

A. Analysis of cytokine and transcription factor on $CD8^+$ and $CD4^+$ T cell from visceral adipose tissue of Flow cytometric analysis *Slc16a1*$^{f/f}$ and *Slc16a1*$^{f/f}$*Tcell*$^{cre}$ mice fed a HFD for 20 weeks; n=11-12 per group.

B. TNF-alpha levels quantified by ELISA assay in serum and epiWAT of *Slc16a1*$^{f/f}$ and *Slc16a1*$^{f/f}$*Tcell*$^{cre}$ mice fed a HFD for 20 weeks; n=10 per group.
C. T cell profile in epiWAT of *Slc16a1*$^{f/f}$ mice fed a SFD or a HFD fed mice; n=3 SFD, n=10 HFD.
D. T cell profile in SCAT of *Slc16a1*$^{f/f}$ mice fed a SFD or a HFD fed mice; n=3 SFD, n=10 HFD.
E,F. Cell/g of dead CD8$^{+}$ T cells (positive for aqua staining) in epiWAT (E) or SCAT (F) of *Slc16a1*$^{f/f}$ and *Slc16a1*$^{f/f}$*Tcell*$^{cre}$ mice fed a HFD for 20 weeks; n=11-12 per group.
epiWAT, epididimal white adipose tissue; FSC, Fluorescence correlation spectroscopy; HFD, high fat diet; SCAT, subcutaneous adipose tissue; SSC, side-scattered light; Slc16a1, solute carrier family 16 member 1.

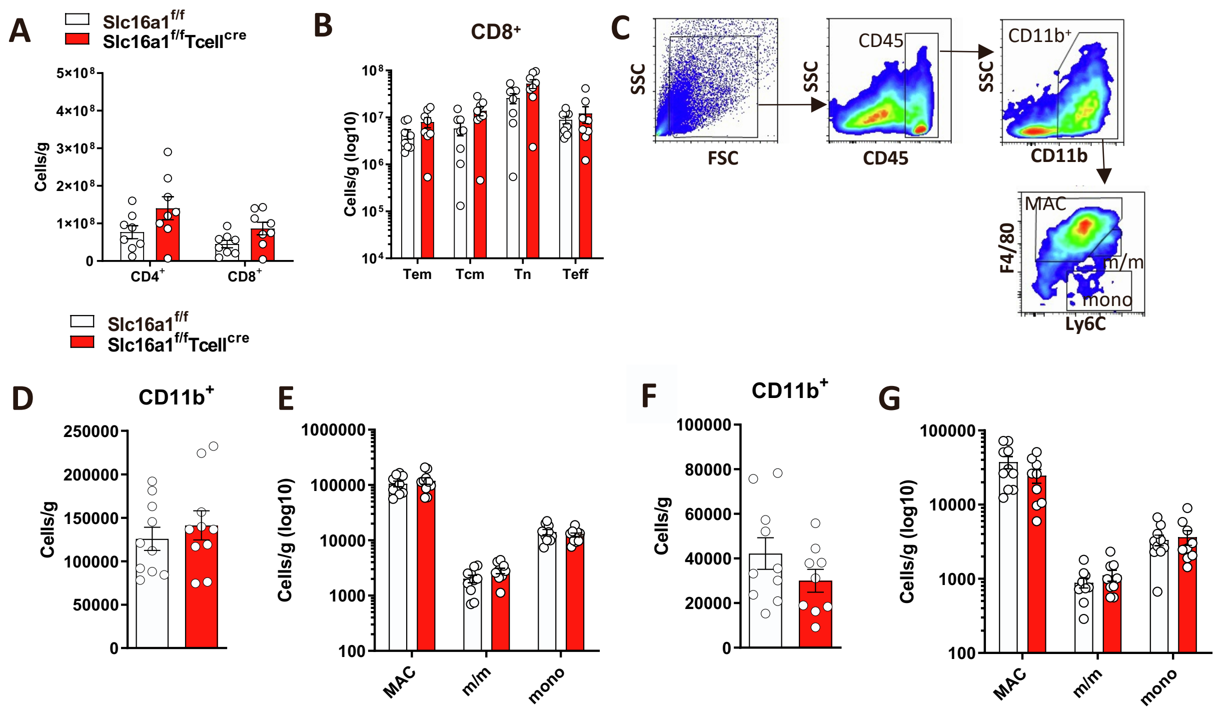


**Figure S6. [Immuno-metabolic signature of obese mice selectively lacking MCT1 in T cells], Related to Figure 6.**

A,B. T lymphocytes ($CD4^+$, $CD8^+$) per gram of tissue (A), and $CD8^+$ subpopulation per gram of tissue (B) in mesenteric lymph nodes of *$Slc16a1^{f/f}$* and *$Slc16a1^{f/f}Tcell^{cre}$* mice fed a HFD for 20 weeks; n=8-10 per group.

C. Gating strategy for innate immune cells staining in epiWAT and SCAT (MAC=macrophages, mono=monocytes, m-m=monocites/macrophages).

D-G. Innate immune cells per gram of tissue in epiWAT (D-E) and SCAT (F-G) of *$Slc16a1^{f/f}$* and *$Slc16a1^{f/f}Tcell^{cre}$* mice fed a HFD for 20 weeks; n=9-10 per group.

In (A-B, D-G) data are presented as mean ± SEM. Differences between groups have been assessed by unpaired two-sides T-test or by two-way ANOVA. $^{**}p < 0.01$ *versus* respective control.
